# NIR-II-triggered plasmonic catalysis with tip-localized enhancement: a strategy for hypoxic biofilm eradication on orthopedic implants

**DOI:** 10.1038/s41377-026-02279-5

**Published:** 2026-04-17

**Authors:** Yu Sun, Fanglin Sheng, Yi Liang, Jinhui Meng, Ke Huang, Sanmao Liu, Yingfeng Qin, Maolin He, Jin-Wen Liu

**Affiliations:** 1 Division of Spinal Surgery, The First Affiliated Hospital of Guangxi Medical University, Nanning, 530021 China; 2 Key Laboratory of Longevity and Aging-related Diseases of Chinese Ministry of Education, Guangxi Colleges and Universities Key Laboratory of Biological Molecular Medicine Research, School of Basic Medical Sciences, Guangxi Medical University, Nanning, 530021 China

**Keywords:** Imaging and sensing, Adaptive optics

## Abstract

The utilization of light as an external stimulus to promote the generation of hot electrons in plasmonic heterostructures and thus augment their catalytic efficacy presents significant potential for bacterial biofilm eradication. However, the inefficient harnessing of photoinduced hot electrons in conventional developed strategies greatly impede their therapeutic application in bone tissues. To overcome these challenges, we herein engineered a near-infrared II (NIR-II)-triggered plasmonic catalysis, which was fabricated through the integration of gold nanobipyramids (Au NBPs) with tip-deposited platinum nanoparticles (Pt NPs), for effective elimination of hypoxic bacterial biofilms on bone implants. The strategic deposition of Pt NPs at the tip of Au NBPs (ePt-Au NBPs) not only brought the redshift of the NIR absorption peak, but also accelerated charge separation and electromagnetic field localization, which endowed the ePt-Au NBPs plasmonic heterostructures with enhanced catalytic activity. Under NIR-II laser irradiation, the plasmonic catalysis with tip-localized enhancement enabled robust generation of hydroxyl radicals (•OH), thereby facilitating the cleavage of extracellular DNA (eDNA) within biofilms, disrupting biofilm integrity, and ultimately sensitizing bacteria to thermal ablation. These attributes collectively contribute to the effective elimination of hypoxic bacterial biofilms. Furthermore, surface functionalization with RGDC peptides conferred the implant with superior biocompatibility and osteogenic integration capabilities. This rationally designed plasmonic catalysis, combining the NIR-II-triggered simultaneous production of enhanced catalytic activity and localized hyperthermia, demonstrates significant potential for translational applications in light-responsive therapeutic strategies for implant-associated infections.

## Abstract (web summary)

We present a rationally designed NIR-II-triggered plasmonic catalysis by strategically depositing platinum nanoparticles onto both tips of gold nanobipyramids, yielding symmetric biconical heterostructures for advanced antimicrobial therapy.

## Graphical abstract

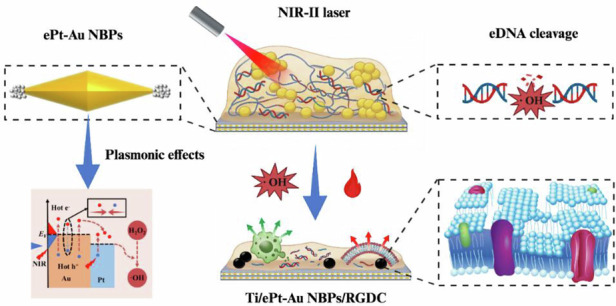

## Introduction

Biofilms, the three-dimensional (3D) microbial communities enveloped within a self-secreted extracellular polymeric substance (EPS) matrix^1,2^, easily colonize on the surfaces of natural organs (teeth, bone, and urethra, etc.) or implanted devices (catheter, stent, and contact lens, etc.) in the human body, leading to persistent infections and therapeutic failures^3–5^. The EPS matrix, composed of polysaccharides, proteins, and extracellular DNA (eDNA), not only fortifies the mechanical integrity of biofilms but also acts as a formidable barrier, impeding immune cell infiltration and reducing antimicrobial agent penetration^6,7^. This dual function exacerbates bacterial resistance to conventional antibiotics, mechanical stress, oxidative stress, and thermal effects more than planktonic bacteria, necessitating innovative strategies to disrupt biofilm viability and eradicate infections.

Current strategies for antibiofilm therapy are mainly categorized into passive and active approaches^8^. Passive strategies, such as surface modifications with antimicrobial coatings or nanostructured topographies, aim to prevent biofilm formation but lack efficacy against already-formed biofilms^9–11^. Besides, the risks of bacterial infection may still occur over subsequent years due to gradual invalidation of the antimicrobial coatings^12,13^. Subsequent biofilm treatment necessitates repeated surgeries and cumbersome therapeutic interventions, resulting in substantial expenditures and compromised clinical outcomes. Conversely, active methodologies target established biofilms, predominantly relying on high-dose antimicrobial agents^14^. While effective in the short term, this approach risks accelerating antimicrobial resistance―a critical limitation in clinical practice^15,16^. In addition, the antibiofilm efficiency is usually low, as it is difficult for antimicrobial agents to penetrate the dense EPS matrix to reach the entrenched microbial populations. Addressing these challenges will require the development of new methods that combine enhanced biofilm penetration with site-specific efficacy, ultimately minimizing antimicrobial resistance evolution and improving clinical outcomes.

Recently, nanosized catalysis, also known as nanozymes, has emerged as a novel antibiotic-free antibacterial agent, leveraging their catalytic efficiency to generate reactive oxygen species (ROS) for biofilm elimination^17–20^. The antibacterial implementation depends on the high enzyme-like catalytic efficiency. To enhance their therapeutic efficacy, maximizing enzyme-like activity through synergistic modulation of physicochemical properties remains a critical focus. Previous studies have indicated that adjusting the physicochemical parameters of nanozymes or introducing external stimuli can improve the catalytic activity of nanozymes^21,22^. Light, as an external stimulus, has emerged as a promising method for enzyme activity regulation due to its non-invasiveness, high temporal and spatial resolution^23^. For instance, carbon-based nanostructures exhibit light-activated oxidase-like behavior via photocatalytic mechanisms^24^, while photosensitized metal-organic frameworks (MOFs) demonstrate wavelength-dependent oxidase-mimicking functionalities for biosensing applications^25^. Such studies underscore the profound influence of photonic energy on augmenting catalytic performance, thereby amplifying antibacterial outcomes. Nevertheless, current light-responsive nanozymes are predominantly constrained in ultraviolet (UV) and visible (Vis) light, which hinders their antimicrobial application in bone tissues^26^. Furthermore, the mechanistic understanding of photo-triggered catalytic pathways and their bactericidal efficacy remains fragmented, impeding the rational design of broad-spectrum and highly efficient antimicrobial platforms.

Localized surface plasmon resonance (LSPR) is a promising approach to enhance enzyme-like activity of nanozymes in catalytic reactions^27,28^. A representative demonstration involves gold nanoparticles (Au NPs), where LSPR-induced hot electron generation under appropriate light irradiation has been shown to remarkably enhance peroxidase-like (POD-like) catalytic efficiency^29^. However, the ultrafast recombination between electron-hole pairs in pure Au NPs results in low utilization of light-induced hot electrons^30^. Moreover, near-infrared (NIR) light, especially near-infrared II (NIR-II) light, is often the better adoption, owing to the reduced image blurring and photothermal conversion efficiency^31–33^. Unfortunately, there are few studies about the NIR-II plasmonic-triggered nanozyme and their applications in bacterial biofilm eradication. Usually, the incorporation of structural complexity into LSPR nanostructures can provide more possibilities for controlling, improving, and broadening their functionality, which are not available in pure and regular counterparts^34,35^. Inspired by the previous works, we proposed a NIR-II plasmonic-enhanced nanozyme catalysis and photothermal performance for bone-implant antibiofilm treatment.

**Scheme 1:**
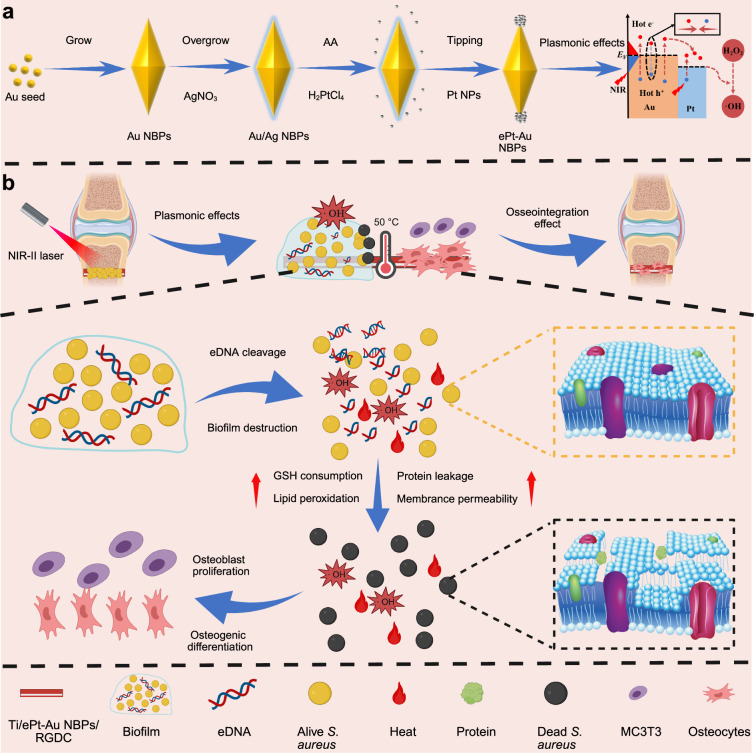
Ti/ePt-Au NBPs/RGDC was engineered for hypoxic bacterial biofilm eradication and bone repair. **a** Synthetic route of ePt-Au NBPs and possible photocatalytic mechanism. **b** The process of bacterial biofilm eradication with NIR-II light irradiation and osteogenic differentiation mediated by RGDC

Herein, we present a rationally designed NIR-II-triggered plasmonic catalysis by strategically depositing platinum nanoparticles (Pt NPs) onto both tips of gold nanobipyramids (Au NBPs), yielding symmetric biconical heterostructures (denoted as ePt-Au NBPs) for advanced antimicrobial therapy. The tip-selective modification of Pt NPs serves dual functions: (1) inducing a pronounced redshift of the LSPR peak into the NIR-II window, and reducing image blurring due to lower scattering; (2) enhancing the spatial separation efficiency of photogenerated electron-hole pairs within Au NBPs, and facilitating the transfer of hot electrons from Au NBPs to adjacent Pt NPs, which effectively suppresses charge recombination and prolongs the lifetime of photoinduced charge carriers. Furthermore, the strong electromagnetic (EM) field localized at the tips of Au NBPs imparts additional hot electron generation to the Pt NPs, collectively augmenting the POD-like activity and photothermal performance of the ePt-Au NBPs (Scheme 1a). Under NIR-II laser irradiation, the plasmonic catalysis with tip-localized enhancement, where the electromagnetic field is significantly enhanced and localized at the tip geometry of nanostructures, enabled robust generation of hydroxyl radicals (•OH), thereby facilitating the cleavage of eDNA within biofilms, disrupting biofilm integrity, and ultimately sensitizing bacteria to thermal ablation. Excitingly, even under hypoxic conditions, the synergistic effects of NIR-II plasmonic-enhanced POD-like activity and localized hyperthermia could effectively kill bacteria through the consumption of intracellular glutathione (GSH), acceleration of lipid peroxidation, disruption of cell membrane integrity, and induction of protein leakage (Scheme 1b). To optimize osseointegration, the nanoengineered ePt-Au NBPs were immobilized on titanium (Ti) bone implants via electrostatic surface self-assembly and further functionalized with RGDC peptides to form Ti/ePt-Au NBPs/RGDC. This surface modification confers the developed plasmonic catalysis with superior biocompatibility and osteogenic activity, ensuring seamless integration with bone implants. Our findings demonstrate that the NIR-II-triggered plasmonic catalysis with tip-localized enhancement holds great promise for non-invasive, hypoxia-tolerant biofilm elimination on orthopedic implants, bridging advanced antimicrobial strategies with functional bone repair. Additionally, the proposed strategy holds tremendous promise for developing next-generation smart coatings to combat implant-associated biofilm infections while fostering bone regeneration.

## Results

### Synthesis and characterization of Au NBPs and ePt-Au NBPs

**Fig. 1:**
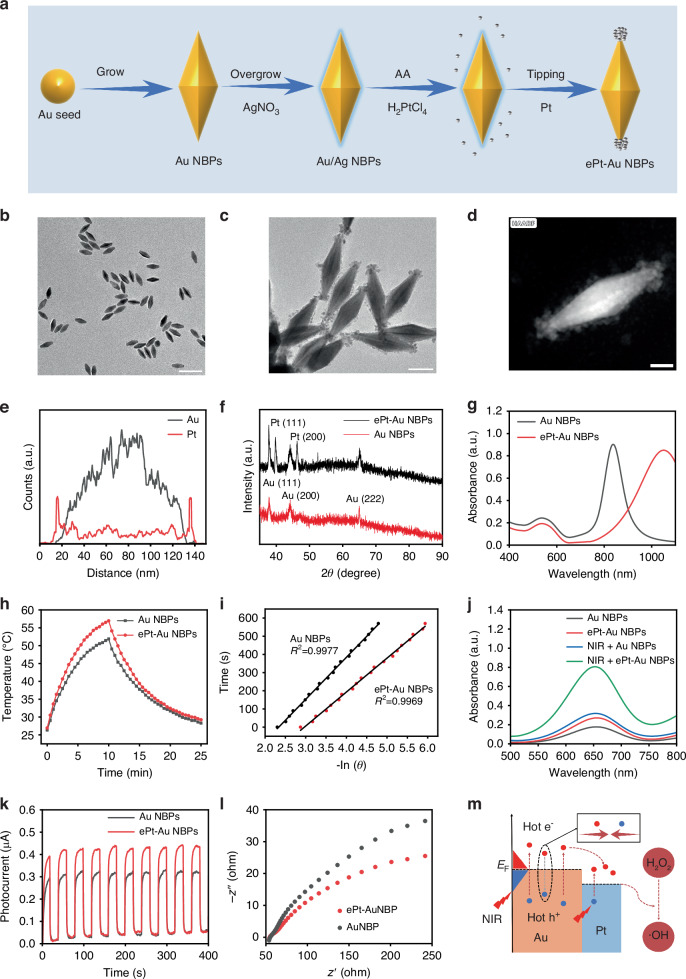
Synthesis, characterization, photothermal performance, and catalytic activity of ePt-Au NBPs (0.6 W cm^−2^). **a** Synthetic route of ePt-Au NBPs. **b** TEM image of Au NBPs (scale bar: 200 nm). **c** TEM image of ePt-Au NBPs (scale bar: 50 nm). **d** HAADF image of ePt-Au NBPs (scale bar: 20 nm). **e** EDS element line scan along the long axis of ePt-Au NBPs. **f** XRD patterns of Au NBPs and ePt-Au NBPs. **g** UV-Vis-NIR absorption spectra of Au NBPs and ePt-Au NBPs. **h** Temperature elevation profiles of Au NBPs and ePt-Au NBPs solution under NIR-II laser irradiation. **i** Photothermal conversion efficiency (η) of Au NBPs and ePt-Au NBPs. **j** POD-like activity of Au NBPs and ePt-Au NBPs with or without NIR-II laser irradiation. **k** Photoelectric current response of Au NBPs and ePt-Au NBPs under NIR-II laser irradiation. **l** EIS spectra of Au NBPs and ePt-Au NBPs. **m** Schematic illustration of the NIR-II enhanced catalytic mechanism of ePt-Au NBPs

The synthesis and structural evolution of Au NBPs and ePt-Au NBPs are illustrated in Fig. 1a. Initially, a crude product containing Au nanospheres and Au NBPs was synthesized via the seed growth method (Figs. S1 and S2). Subsequently, purification and separation of Au NBPs from the Au nanospheres were achieved through depletion flocculation using benzyldimethylhexadecylammonium chloride (BDAC) as the surfactant. Transmission electron microscope (TEM) images revealed that the as-prepared Au NBPs possessed a biconical structure with a length of 115.02 ± 11.40 nm and a diameter of 26.35 ± 4.12 nm (Figs. 1b and S3). Following the synthesis of Au NBPs, a thin silver layer was epitaxially deposited onto the Au NBP surfaces to facilitate end-specific Pt NPs attachment, ultimately forming symmetrical ePt-Au NBPs. TEM imaging demonstrated that Pt NPs (2.76 ± 0.23 nm in diameter) mainly deposited at the tips of the Au NBPs (Figs. 1c and S4). To further elucidate the spatial distribution of Pt NPs, high-angle annular dark-field scanning transmission electron microscopy (HAADF-STEM) was employed. The HAADF-STEM images and corresponding elemental mapping corroborated the tip-selective deposition of Pt NPs (Fig. 1d, e), aligning with the TEM observations. High-resolution TEM (HRTEM) images of Au NBPs exhibited the lattice spacing at 0.235 nm and 0.20 nm, corresponding to the (111) and (200) planes of Au, respectively (Fig. S5). For ePt-Au NBPs, HRTEM imaging identified a lattice spacing of 0.224 nm, consistent with the (111) plane of Pt (Fig. S6), confirming the crystalline integrity of both components. X-ray diffraction (XRD) analysis further validated that Au NBPs exhibited characteristic diffraction peaks of Au at 38.2°, 44.3°, and 64.6°, while ePt-Au NBPs displayed additional Pt-specific peaks at 39.9° and 46.5° (Fig. 1f), which further verified the attachment of Pt NPs onto Au NBPs.

In addition, zeta potential measurements revealed a significant increase in surface charge from 19.57 ± 1.62 mV for Au NBPs to 39.73 ± 1.45 mV for ePt-Au NBPs (Fig. S7), indicative of enhanced colloidal stability post-Pt NPs deposition. The optical properties of both materials were investigated using UV-Vis-NIR spectroscopy. As shown in Fig. 1g, Au NBPs exhibited a pronounced longitudinal LSPR peak at 839 nm and a weak transverse plasmonic absorption at 525 nm. After the end of deposition of Pt NPs, the LSPR peak of ePt-Au NBPs underwent a substantial redshift to 1050 nm within the NIR-II window, while the transverse absorption remained almost unchanged. It is worth noting that the light scattering coefficient in the NIR-II region (1000–1350 nm) is significantly lower than that in NIR-I (650–950 nm), leading to reduced image blurring during propagation in biological tissues. Although the absorption of water increases gradually in the NIR-II region, which limits the absolute penetration depth compared with NIR-I, the lower scattering enables more accurate light delivery to orthopedic implant sites^36^. This feature is critical for activating plasmonic catalysis and photothermal effects on the implant surface while minimizing nonspecific heating of superficial tissues.

### The photothermal and photocatalytic performance of ePt-Au NBPs

As mentioned above, the synthesized ePt-Au NBPs exhibited exceptional NIR-II absorption characteristics (Fig. 1g), highlighting their potential as excellent photothermal agents. Upon NIR-II laser irradiation for 10 min, the solution temperatures of Au NBPs and ePt-Au NBPs rose by 24.5 °C and 29.5 °C, respectively (Fig. S8), demonstrating the superior photothermal response of the latter. Quantitative analysis of photothermal conversion efficiencies (η), calculated from heating-cooling curves, revealed values of ~50% for Au NBPs and ~64% for ePt-Au NBPs, which exhibit superior photothermal conversion efficiency compared with other common NIR-responsive materials^20^ (Fig. 1h, i). This significant η enhancement for ePt-Au NBPs is attributed to the synergistic effect between the plasmonic Au NBPs and Pt NPs at the end tips of the bipyramid, which optimizes electron dynamics and suppresses electron carrier recombination. Furthermore, systematic investigations confirmed that increasing ePt-Au NBPs concentration (0–160 μg mL^−1^) or laser power density (0–0.6 W cm^−2^) induced a proportional temperature elevation (Figs. S9 and S10).

The POD-like catalytic activity of both materials was evaluated via oxidation of 3,3′,5,5′-tetramethylbenzidine (TMB) in the presence of hydrogen peroxide (H_2_O_2_). Under dark conditions, minimal catalytic activity was observed for Au NBPs and ePt-Au NBPs. However, NIR-II irradiation triggered a dramatic increase in activity, consistent with the Arrhenius principle that thermal energy accelerates catalytic kinetics, and with ePt-Au NBPs outperforming Au NBPs by a substantial margin (Fig. 1j), which is attributed to the superior LSPR performance of ePt-Au NBPs. Parameter optimization studies for ePt-Au NBPs revealed that catalytic efficiency correlated positively with material concentration (0–160 μg mL^−1^), irradiation time (0–15 min), temperature (25–50 °C), and laser power (0–0.6 W cm^−2^) (Fig. S11a–d). Notably, pH (pH 4–8) inspection disclosed that alkaline conditions gradually diminished POD-like activity of ePt-Au NBPs (Fig. S11e), suggesting proton-dependent catalytic pathways. Steady-state kinetic analyses under NIR-II irradiation revealed higher maximum reaction velocities (*V*_max_) and lower Michaelis constants (*K*_m_) for ePt-Au NBPs compared to non-irradiated systems (Fig. S12), indicating NIR-II plasmonic-enhanced substrate affinity and catalytic turnover. Remarkably, ePt-Au NBPs retained structural integrity, photothermal, photoabsorption, and catalytic performance over five consecutive irradiation-cooling cycles (Fig. S13), underscoring their exceptional stability for practical applications.

Plasmonic gold nanostructures, typical examples of which are gold nanorods (Au NRs), exhibit longitudinal LSPR, enabling efficient NIR light absorption. Finite-difference time-domain (FDTD) simulations revealed that Au NBPs generate stronger EM fields with a maximum |*E* | ^2^/ | *E*_0_ | ^2^ value of 150 than Au NRs with a maximum |*E* | ^2^/ | *E*_0_ | ^2^ value of only 10.1 (Fig. S14), facilitating stronger hot electron generation. However, rapid electron-phonon scattering in noble metals often hampers hot electron utilization^30^. To address this, Pt NPs specific integration at the end tips of Au NBP (i.e., ePt-Au NBPs) was engineered to enhance hot electron separation and localization. FDTD simulations further demonstrated that ePt-Au NBPs exhibit intensified EM fields (|*E* | ^2^/ | *E*_0_ | ^2^ value of 136) compared to all-deposited Pt counterparts (|*E* | ^2^/ | *E*_0_ | ^2^ value of 108) (aPt-Au NBPs, Fig. S15), which may be attributed to the fact that full coverage of Pt NP layers on the surfaces of Au NBP attenuates the light absorption. Meanwhile, we observed that ePt-Au NBPs showed a higher photothermal and photocatalytic activity compared with aPt-Au NBPs (Fig. S16). Photo-electrochemical analyses also corroborated these findings. As shown in Fig. 1k, compared to Au NBPs, ePt-Au NBPs displayed higher photocurrent density, implying stronger plasmon-exciton coupling after Pt NPs deposition at the tips of Au NBPs, and simultaneously, reduced electrochemical impedance *arc* radius (Fig. 1l), indicative of efficient and fast hot electron transfer and minimized charge recombination.

Based on these results, a possible photocatalytic mechanism was proposed (Fig. 1m). Under NIR-II excitation, plasmon-induced hot electrons on Au NBPs migrate toward Pt tips, so that the excited hot electrons can be effectively captured into the conduction band of Pt NPs, thereby achieving spatial separation of electrons and holes and avoiding rapid recombination within Au NBPs. Concurrently, the strong EM field at the bipyramidal tips of Au NBPs enable to endow Pt NPs with additional hot electrons generation capability, augmenting their intrinsic catalytic activity. This dual mechanism—enhanced charge separation and EM field localization—synergistically elevates the photocatalytic performance of ePt-Au NBPs, positioning them as versatile platforms for NIR-II-driven biomedical applications.

### Preparation and characterization of Ti/ePt-Au NBPs/RGDC implants

**Fig. 2:**
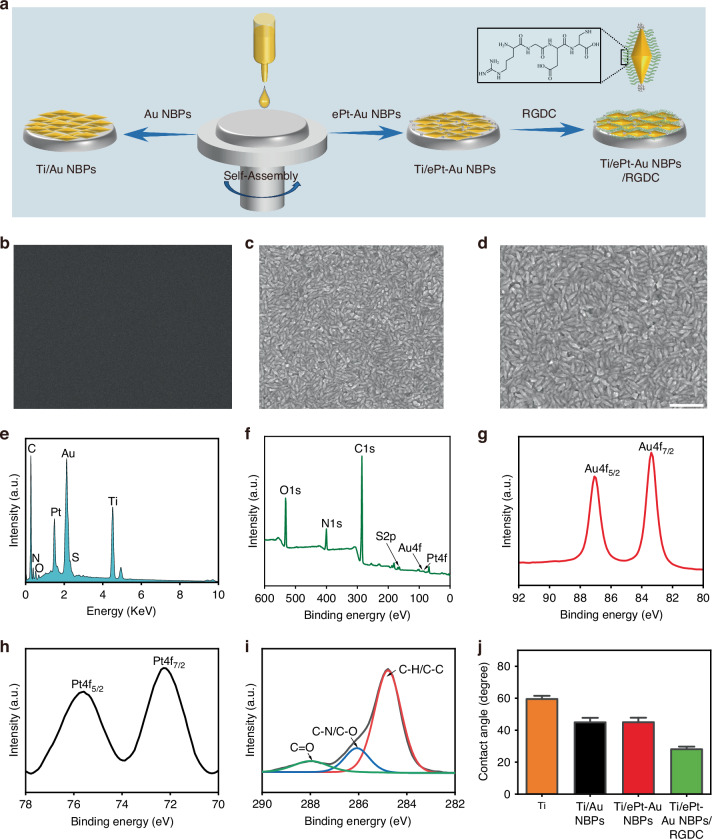
Synthesis and characterization of Ti/ePt-Au NBPs/RGDC. **a** Synthetic route of Ti/ePt-Au NBPs/RGDC. **b** SEM image of bare Ti. **c** SEM image of Ti/ePt-Au NBPs. **d** SEM image of Ti/ePt-Au NBPs /RGDC (scale bar: 500 nm). **e** EDS spectrum of Ti/ePt-Au NBPs /RGDC. **f** XPS spectra of Ti/ePt-Au NBPs/RGDC. **g** Au 4f spectrum. **h** Pt 4f spectrum. **i** C 1s spectrum. **j** Contact angles of different Ti implants

The Ti/ePt-Au NBPs implants were fabricated via an electrostatic surface self-assembly technique, wherein engineered ePt-Au NBPs were uniformly deposited onto Ti substrates (Fig. 2a). Notably, this surface modification still preserved the intrinsic morphology of the ePt-Au NBPs, as confirmed by scanning electron microscopy (SEM) (Fig. 2b, c). Moreover, SEM imaging revealed a homogeneously distributed ePt-Au NBP coating on the Ti surface, with no observable aggregation or structural deformation compared to unmodified Ti. RGDC peptide, a cell-adhesive and proliferation motif that accelerates tissue regeneration^37^, was subsequently functionalized via a strong Au-S bond to yield the final Ti/ePt-Au NBPs/RGDC interface. SEM analysis after RGDC modification showed no significant change in the morphology of the ePt-Au NBP (Fig. 2d), emphasizing the stability of the ePt-Au NBPs under peptide conjugation conditions.

Elemental composition and chemical states were systematically interrogated using an energy-dispersive spectrometer (EDS) and X-ray photoelectron spectroscopy (XPS). EDS mapping confirmed the coexistence of Ti, Au, Pt, C, N, O, and S on the Ti/ePt-Au NBPs/RGDC surface (Fig. 2e), corroborating the successful integration of ePt-Au NBPs and RGDC on the Ti substrates. As expected, XPS survey spectra identified characteristic peaks for Au 4f, Pt 4f, C 1s, N 1s, O 1s, and S 2p (Fig. 2f). Deconvolution of high-resolution Au 4f spectra resolved two distinct doublets at 83.9 eV (Au 4f_7/2_) and 87.6 eV (Au 4f_5/2_), indicative of metallic Au^0^ (Fig. 2g). Similarly, Pt 4f peaks at 71.0 eV (Pt 4f_7/2_) and 74.5 eV (Pt 4f_5/2_) confirmed the presence of metallic Pt^0^, consistent with the engineered decoration of Pt NPs on Au NBPs (Fig. 2h). Critical insights into the high-resolution XPS spectral for C1s analysis (Fig. 2i), which can be fitted into three components at 284.4 eV (C–H/C–C, hydrocarbon backbone), 286.2 eV (C–N/C–O, peptide linkages and hydroxyl groups), and 288.1 eV (C=O, carbonyl moieties). These findings clearly validated that ePt-Au NBPs and RGDC peptide were successfully loaded onto the Ti surface. The synergistic combination of ePt-Au NBPs and RGDC peptides establishes a multifunctional interface with potential dual functionality, namely, ePt-Au NBPs-mediated biofilm suppression and RGDC-driven cellular adhesion and proliferation.

Hydrophilicity, a critical determinant of biocompatibility, was quantified via contact angle measurements. Progressive surface modifications reduced contact angles from 59.52° (bare Ti) to 28.13° (Ti/ePt-Au NBPs/RGDC) (Fig. 2j). This marked enhancement in hydrophilicity for the RGDC-functionalization aligns with the amphiphilic nature of the peptide, which introduces polar functional groups (e.g., carboxyl and amine) to the surface. The enhanced hydrophilicity is anticipated to promote affinity between cells and implants^38,39^. Taken together, the Ti/ePt-Au NBPs/RGDC nanocomposite combines the biofilm eradication capacity of ePt-Au NBPs nanostructures with the bioactivity of RGDC peptides, offering a promising platform for orthopedic implants.

### The photothermal and photocatalytic performance of Ti/ePt-Au NBPs/RGDC implants

**Fig. 3:**
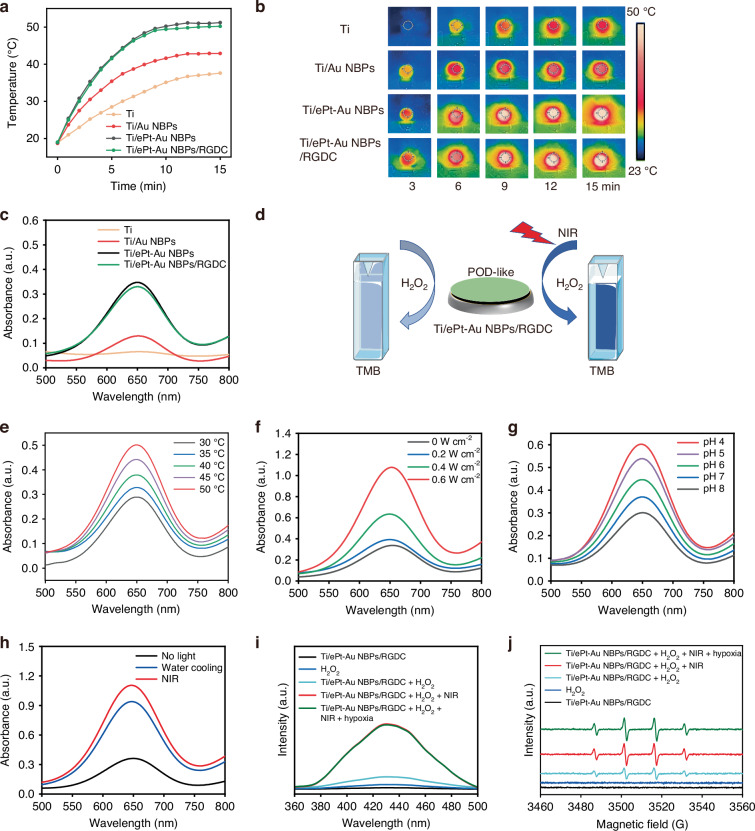
NIR-II-enhanced POD-like activity of Ti/ePt-Au NBPs/RGDC implants (0.6 W cm^−2^). **a** Temperature elevation curves of different Ti implants under NIR-II laser irradiation. **b** Infrared thermal images of different Ti implants under NIR-II laser irradiation. **c** POD-like activity of different Ti implants. **d** Schematic illustration of NIR-II-enhanced POD-like activity of Ti/ePt-Au NBPs/RGDC implants. **e** POD-like activity of Ti/ePt-Au NBPs/RGDC implants at different temperatures. **f** POD-like activity of Ti/ePt-Au NBPs/RGDC implants under different laser intensities. **g** POD-like activity of Ti/ePt-Au NBPs/RGDC at different pH values. **h** Comparison of the effects of photocatalysis and photothermal effects on catalytic activity. **i** Fluorescence spectra of TA in different treatment groups. **j** ESR spectra in different treatment groups

The integration of NIR-II plasmonic-enhanced nanozymes with photothermal and photocatalytic functionalities offers a promising strategy for combating bacterial biofilms in bone implants. The photothermal behavior of the Ti/ePt-Au NBPs/RGDC implants was evaluated under 1064 nm laser irradiation. As shown in Figs. 3a, b and S17a, the temperature of Ti/ePt-Au NBPs increased to 51.2 °C within 15 min. Due to the insulating effect of RGDC peptide layers, the temperature increase of Ti/ePt-Au NBPs/RGDC (50.2 °C) was slightly lower than that of Ti/ePt-Au NBPs, which was consistent with previous studies^40–42^. Notably, ePt-Au NBPs-based Ti implants exhibited superior photothermal efficiency compared to Ti (37.6 °C) and Ti/Au NBPs (42.9 °C), attributed to the LSPR of ePt-Au NBPs, which efficiently converts NIR-II photons into thermal energy, and the synergistic effect between the plasmonic Au NBPs and Pt NPs at the end tips of the bipyramid, which optimizes electron dynamics and suppresses electron carrier recombination. This photothermal response is critical for eliminating bacterial biofilm and enhancing enzymatic activity.

The POD-like activity of Ti/ePt-Au NBPs/RGDC implants was quantified via the oxidation of TMB, monitored at 652 nm (Figs. 3c and S17b). As observed, the modification of RGDC almost has no effect on the catalytic performance of the implant. Ti/ePt-Au NBPs/RGDC implants demonstrated robust catalytic activity, which was further amplified under NIR-II irradiation (Fig. 3d). Temperature-dependent studies revealed a ~1.75 fold increase in activity between 30 °C and 50 °C (Figs. 3e and S18a), aligning with Arrhenius kinetics and confirming thermal acceleration of enzymatic reactions^43^. Besides, Ti/ePt-Au NBPs/RGDC implants were found to have an increasing catalytic activity with increasing light intensity in the range of 0–0.6 W cm^−2^ (Figs. 3f and S18b). Intriguingly, the POD-like had high catalytic efficiency at slightly acidic conditions (Figs. 3g and S18c), matching the microenvironment of bacterial infections (pH 5.5–6.5, elevated H_2_O_2_ levels), thus highlighting the suitability of Ti/ePt-Au NBPs/RGDC implants for in situ applications.

Further, control experiments were conducted to investigate the contributions of photothermal and photocatalytic effects for enhanced •OH generation (Fig. 3h). Both thermal energy and hot electrons that generated via LSPR were found to enhance peroxidase-like activity, with hot electron carriers contributing ~70% of the total catalytic enhancement (Fig. S19). This exploration of POD-like activity mechanism was further performed by hydroxyl radical (•OH) detection using terephthalic acid (TA)-based fluorescence assays and 5, 5-dimethyl N-oxide pyrroline (DMPO) trapped electron spin resonance (ESR) spectroscopy. Under NIR-II irradiation, the Ti/ePt-Au NBPs/RGDC + H_2_O_2_ group exhibited ~5.2-fold increase in TAOH (TA oxidation product) fluorescence intensity (Figs. 3i and S20) and intensified DMPO-•OH ESR signal (Fig. 3j), confirming NIR-II triggered •OH enhancement via H_2_O_2_ decomposition. Moreover, the Ti/ePt-Au NBPs/RGDC implants demonstrated remarkable stability, retaining >90% catalytic activity after five irradiation cycles (Fig. S21), which positions them as a potent therapeutic platform for implant-associated bacterial biofilm eradication.

### In vitro antibacterial activity

**Fig. 4:**
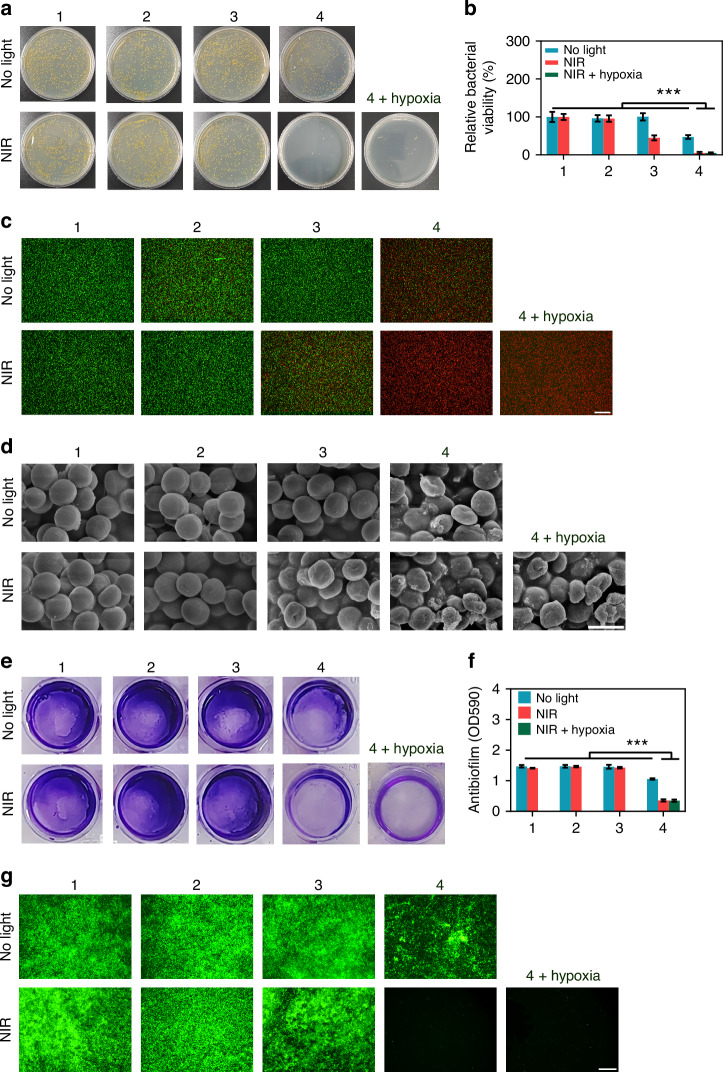
In vitro antibacterial and biofilm eradication activities of Ti/ePt-Au NBPs/RGDC (0.6 W cm^−2^). **a** Photographs of bacterial colonies in different treatment groups. **b** Viability of bacteria in different treatment groups. **c** Live/dead staining of bacteria in different treatment groups (scale bar: 100 μm). **d** SEM images of bacteria in different treatment groups (scale bar: 1 μm). **e** Crystal violet staining of bacterial biofilms in different treatment groups. **f** Quantification of biofilm biomass in different treatment groups. **g** Live/dead staining of bacterial biofilms in different treatment groups (scale bar: 100 μm). (1) Ti, (2) Ti + H_2_O_2_, (3) Ti/ePt-Au NBPs/RGDC, (4) Ti/ePt-Au NBPs/RGDC + H_2_O_2_. (*p < 0.05, **p < 0.01, and ***p < 0.001)

Leveraging the synergistic photothermal and catalytic properties of the engineered Ti/ePt-Au NBPs/RGDC plasmonic catalysis, we systematically evaluated its potential for combating bacterial infections associated with biomedical implants. As illustrated in Fig. 4a, b, control groups (Ti and Ti + H_2_O_2_) exhibited negligible antibacterial activity under both NIR-II irradiation and dark conditions, with dense bacterial colonies proliferating across the agar plates. Similarly, the Ti/ePt-Au NBPs/RGDC group without irradiation displayed no significant bactericidal effect, verifying the light-dependent activation of its antibacterial functionalities. However, upon NIR-II exposure, a dramatic reduction in bacterial colonies was observed for the Ti/ePt-Au NBPs/RGDC group, attributable to localized hyperthermia generated via plasmonic photothermal conversion.

The combinatorial application of Ti/ePt-Au NBPs/RGDC with H_2_O_2_ further amplified antibacterial efficacy, as evidenced by diminished bacterial colonies in the Ti/ePt-Au NBPs/RGDC + H_2_O_2_ group (Fig. 4a, b). Briefly, without NIR-II laser irradiation, the Ti/ePt-Au NBPs/RGDC + H_2_O_2_ group exhibited a reduced number of colonies compared to the other group, suggesting that the antibacterial activity mainly stems from the catalytic activity of Ti/ePt-Au NBPs/RGDC. However, the photothermal effect or POD-like activity of Ti/ePt-Au NBPs/RGDC alone can only eliminate approximately 50% of the bacteria, which does not meet the sterilization requirements of clinical implants. After NIR-II laser irradiation, a tremendous reduction in bacterial colonies was observed. This phenomenon underscored the catalytic generation of •OH through POD-like activity, which synergized with photothermal effects under NIR-II irradiation to achieve near-complete bacterial eradication. Complementary live/dead staining and SEM imaging corroborated these findings, revealing extensive membrane disruption and structural collapse in bacteria treated with this dual-modal therapeutic strategy (Fig. 4c, d).

Biofilms, notorious for conferring antibiotic resistance, were visualized and quantitatively assessed via crystal violet staining (Fig. 4e, f). The Ti/ePt-Au NBPs/RGDC + H_2_O_2_ + NIR group demonstrated a remarkable reduction in biofilm biomass compared to control groups, outperforming both only photothermal (NIR + Ti/ePt-Au NBPs/RGDC) and catalytic (Ti/ePt-Au NBPs/RGDC + H_2_O_2_) treatments. This result highlighted the critical role of concurrent •OH generation and hyperthermia (~50 °C, Fig. S17a) in destabilizing biofilm matrices. Noted that, without NIR-II laser irradiation, the Ti/ePt-Au NBPs /RGDC + H_2_O_2_ group also showed a reduced content of crystal violet, indicating that the •OH generated by the catalysis of Ti/ePt-Au NBPs /RGDC was enough to partially disrupt the pre-formed biofilms. Previous studies have reported that a temperature of 70 °C can eliminate biofilms^44,45^. Meanwhile, it was found that moderate photothermal effects (~50 °C) alone failed to disrupt biofilms (Fig. 4e, f), which were further confirmed by bacterial live/dead staining (Fig. 4g). Excitingly, even under hypoxic conditions, a hallmark of chronic infections, the Ti/ePt-Au NBPs/RGDC + H_2_O_2_ + NIR group retained superior antibacterial efficiency (Fig. 4a–g), suggesting robust catalytic functionality independent of ambient oxygen levels.

Collectively, the developed Ti/ePt-Au NBPs/RGDC can serve as a pioneering NIR-II-triggered plasmonic catalysis capable of dual-modal bacterial eradication through spatially and temporally controlled photothermal-catalytic synergy. The excellent ability of Ti/ePt-Au NBPs/RGDC to function under hypoxia, coupled with its biofilm-penetrating efficacy, addresses critical limitations in current implant infection therapies. The hypoxia-tolerant performance of this system holds particular clinical relevance, as infected implant niches often exhibit low oxygen tension. We posit that the NIR-II plasmonic-enhanced electron transfer at the ePt-Au NBPs interface amplifies H_2_O_2_ activation kinetics, enabling sustained •OH production even under oxygen-deprived conditions, a mechanism distinct from conventional photocatalysts reliant on O_2_-mediated pathways^46^.

### Mechanism of antibacterial activity

To elucidate the antibacterial mechanism of the NIR-II-triggered Ti/ePt-Au NBPs/RGDC plasmonic catalysis, a systematic investigation was conducted to evaluate its multifaceted effects on bacterial membrane integrity, oxidative stress induction, and biofilm disruption. As mentioned above, the synergistic interplay between LSPR-driven photothermal activity and nanozyme-mediated catalysis was identified as the cornerstone of its potent antibacterial performance.

**Fig. 5:**
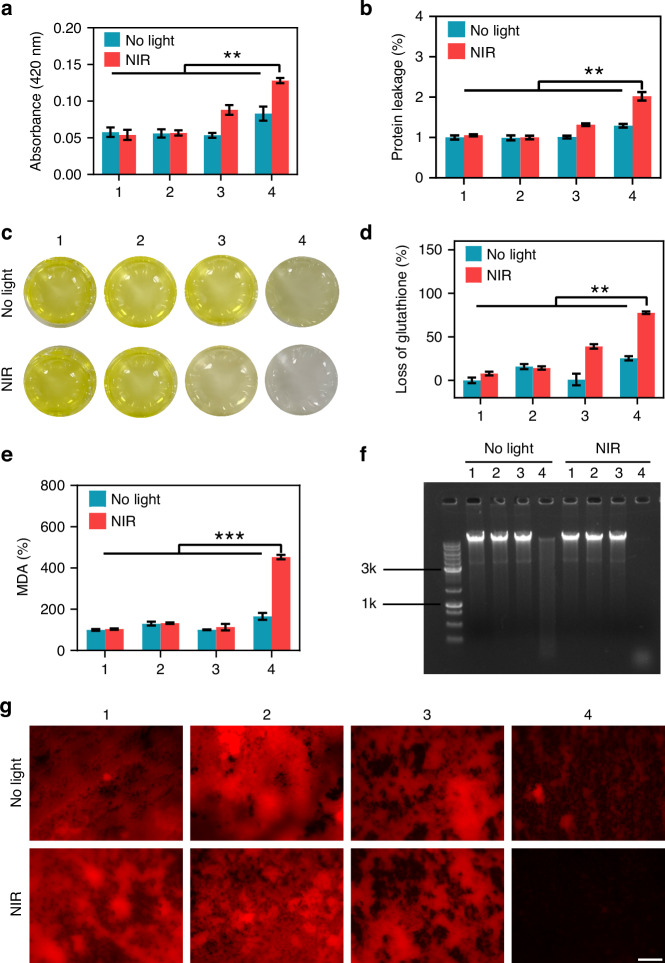
The antibacterial mechanism of Ti/ePt-Au NBPs/RGDC (0.6 W cm^−2^). **a** ONPG hydrolysis assay to evaluate changes in bacterial cell membrane permeability. **b** Bacterial protein leakage assessment. **c** Images showing the color change of the GSH solution in different treatment groups. **d** Quantification of GSH loss. **e** Assessment of bacterial lipid peroxidation levels. **f** Evaluation of bacterial biofilm eDNA degradation. **g** Staining results of bacterial biofilm eDNA (scale bar: 100 μm). (1) Ti, (2) Ti + H_2_O_2_, (3) Ti/ePt-Au NBPs/RGDC, (4) Ti/ePt-Au NBPs/RGDC + H_2_O_2_. (*p < 0.05, **p < 0.01, and ***p < 0.001)

The production of •OH, a highly reactive ROS, was first verified using a 2′,7′-dichlorodihydrofluorescein diacetate (DCFH-DA) fluorescent probe. As anticipated, the NIR + Ti/ePt-Au NBPs/RGDC + H_2_O_2_ group exhibited the most intense fluorescence signal, confirming the generation of a large amount of •OH (Fig. S22). The •OH is known to compromise bacterial membrane integrity and increase its permeability by inducing lipid peroxidation and protein oxidation^47^. To quantify membrane damage, a 2-Nitrophenyl β-D-galactopyranoside (ONPG) hydrolysis assay was performed. The previous results showed that pure Ti implants exhibit only a minimal temperature increase from 19 °C to approximately 38 °C under NIR-II laser irradiation, indicating its weak absorption of 1064 nm photons and inability to generate effective localized thermal effects (Fig. 3). Consequently, no obvious differences between samples 1 and 2 (without or with NIR-II laser) were observed in Fig. 5. Notably, the Ti/ePt-Au NBPs/RGDC + H_2_O_2_ group under NIR-II irradiation demonstrated a 2.3-fold increase in ONPG hydrolysis compared to non-irradiated controls (Fig. 5a), signifying severe membrane permeabilization. This phenomenon arises from the dual action of LSPR-enhanced photothermal effects, which destabilize membrane fluidity, and enhanced catalytic decomposition of H_2_O_2_, which accelerates •OH diffusion. Protein leakage assays further corroborated these findings, with the NIR + Ti/ePt-Au NBPs/RGDC + H_2_O_2_ group showing 2.1-fold higher extracellular protein levels than the Ti control (Fig. 5b), confirming catastrophic membrane disruption.

GSH, a critical intracellular antioxidant, serves as a biomarker for oxidative stress. Ellman’s assay revealed near-complete oxidation of GSH in the NIR + Ti/ePt-Au NBPs/RGDC + H_2_O_2_ group, as evidenced by the decolorization of GSH solutions (Fig. 5c, d). This depletion indicated an overwhelming ROS burden that exhausts bacterial defense mechanisms. Concurrently, lipid peroxidation levels, quantified via malondialdehyde (MDA) assays, surged by 4.7-fold in the synergistic treatment group (Fig. 5e). Such extensive peroxidative damage can disrupt membrane fluidity, ion homeostasis, and ATP synthesis, ultimately leading to bacterial metabolic collapse^48^.

Biofilm integrity relies heavily on eDNA, which stabilizes the extracellular matrix. However, the ROS, especially the highly reactive •OH, can cleave eDNA to disrupt the integrity of biofilms. Gel electrophoresis and hydroxy-9,9-dimethyl-2 (9H)-acridone (DDAO) staining demonstrated that the NIR + Ti/ePt-Au NBPs/RGDC + H_2_O_2_ treatment effectively cleaved eDNA into small fragments (Fig. 5f) and well eliminated fluorescence signals of DDAO-stained eDNA in biofilms (Fig. 5g), indicating the removal of eDNA in biofilms. This degradation is attributed to •OH-mediated cleavage of phosphodiester bonds in eDNA, a process potentiated by plasmonic heating. The latter enhances nanozyme activity and promotes •OH diffusion into biofilm matrices, thereby overcoming the protective barriers of bacterial communities.

### Biocompatibility evaluation of NIR-II-triggered plasmonic catalysis modified with RGDC for bone-implant applications

**Fig. 6:**
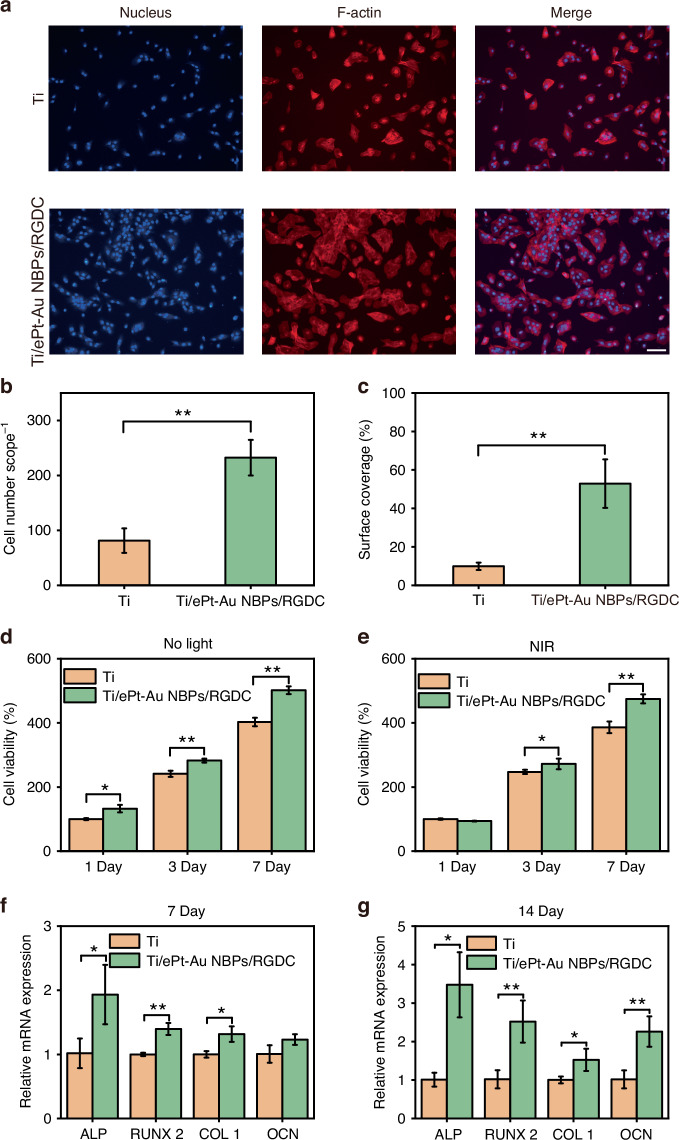
Biocompatibility assessment of Ti/ePt-Au NBPs/RGDC (0.6 W cm^−2^). **a** Morphology of MC3T3-E1 cells after co-culture with implants for 4 h (scale bar: 20 μm). **b** Quantification of cell adhesion number. **c** Quantification of cell adhesion area. **d** Cell proliferation on different implant surfaces measured by CCK-8 assay. **e** Cell proliferation on different implant surfaces after NIR-II laser irradiation measured by CCK-8 assay. **f** Expression of osteogenic-related genes in MC3T3-E1 cells cultured on different sample surfaces for 7 days. **g** Expression of osteogenic-related genes in MC3T3 -E1 cells cultured on different sample surfaces for 14 days. (*p < 0.05, **p < 0.01, and ***p < 0.001)

The adhesion behavior of MC3T3-E1 pre-osteoblasts on Ti/ePt-Au NBPs/RGDC and control Ti surfaces was systematically investigated via fluorescence staining with 4′,6-diamidino-2-phenylindole (DAPI) and rhodamine-phalloidin. As depicted in Figs. 6a–c and S23, the Ti/ePt-Au NBPs/RGDC substrates demonstrated significantly enhanced cell adhesion, evidenced by a higher density of adhered cells and expanded adhesion area at both 4 and 24 h post-seeding compared to Ti alone. To elucidate the molecular mechanism underlying this improvement, Real-time quantitative polymerase chain reaction (RT-qPCR) analysis revealed upregulated expression of *integrin αv* and *β3* subunits in the Ti/ePt-Au NBPs/RGDC group (Fig. S24). These findings can be attributed to the fact that RGDC peptide modification facilitates integrin-mediated cellular adhesion, likely through specific interactions between the RGDC motif and αvβ3 integrin receptors, thereby promoting early-stage cell-material interactions critical for osseointegration.

Cell proliferation dynamics were assessed using the CCK-8 assay over 7 days (Fig. 6d). Notably, while the Ti/ePt-Au NBPs/RGDC group exhibited transiently reduced viability at 1 day under NIR-II laser irradiation (1064 nm, 0.6 W cm^−2^), attributed to localized photothermal effects (Fig. 6e). However, a remarkable recovery and subsequent proliferation advantage were observed at 3 and 7 days. This rebound phenomenon implied that the photothermal stress was transient, and the RGDC-functionalized surface compensates for initial thermal damage by enhancing cellular adhesion, thereby fostering cellular adaptation and sustained proliferation.

To evaluate osteogenic differentiation, alkaline phosphatase (ALP) activity, a hallmark of early osteogenesis, was quantified. The Ti/ePt-Au NBPs/RGDC group displayed significantly elevated ALP levels at 4 and 7 days (Fig. S25), corroborated by enhanced collagen secretion (7 and 14 days, Fig. S26) and extracellular matrix (ECM) mineralization (14 and 21 days, Fig. S27), indicative of progressive osteogenic maturation. RT-qPCR further revealed temporal regulation of key osteogenic differentiation marker genes. At 7 days, ALP, runt-related transcription factor 2 (RUNX 2), and type 1 collagen (COL 1) expressions were markedly upregulated in the

Ti/ePt-Au NBPs/RGDC group compared to Ti (Fig. 6f), while osteocalcin (OCN), a late-stage differentiation marker, showed comparable levels. By 14 days, however, OCN expression increased significantly in the Ti/ePt-Au NBPs/RGDC group (Fig. 6g), which is in good agreement with the observed ECM mineralization. This staged gene activation underscores the role of RGDC in accelerating osteogenic commitment, likely by amplifying integrin-mediated mechanotransduction pathways that drive pre-osteoblasts toward terminal differentiation.

### In vivo antibacterial evaluation of NIR-II-triggered plasmonic catalysis for biofilm eradication in bone implants

**Fig. 7:**
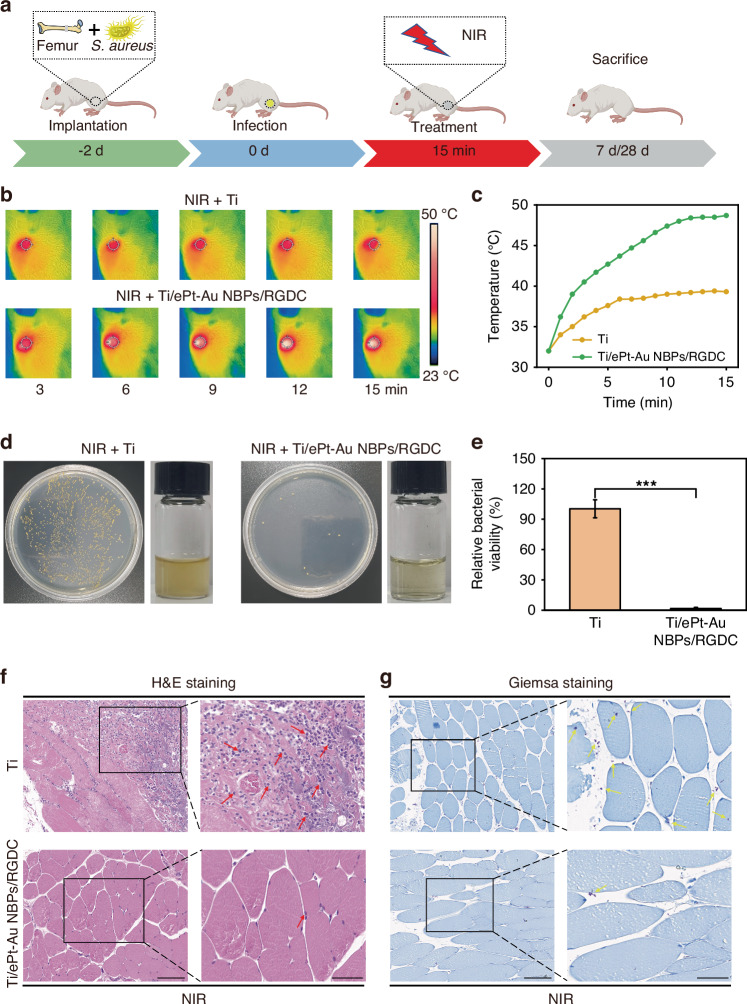
In vivo antibacterial activity of implants (0.6 W cm^−2^). **a** Flowchart of the animal experiment. **b** Real-time thermal imaging photographs of Ti and Ti/ePt-Au NBPs/RGDC implants under NIR-II laser irradiation. **c** Temperature elevation curves of Ti and Ti/ePt-Au NBPs/RGDC implants under NIR-II laser irradiation. **d** Photographs of bacterial colonies and turbidity in different treatment groups. **e** Bacterial viability of different treatment groups. **f** Evaluation of soft tissue infection in different treatment groups using H&E staining. Scale bars: 100 μm (left) and 50 μm (right). Red arrows represent neutrophils. **g** Evaluation of soft tissue infection in different treatment groups using Giemsa staining. Scale bars: 100 μm (left) and 50 μm (right). Yellow arrows represent bacteria (*p < 0.05, **p < 0.01, and ***p < 0.001)

Building upon the outstanding in vitro antibacterial efficacy and biocompatibility of the Ti/ePt-Au NBPs/RGDC implants, we further investigated their antibiofilm and osteogenic potential in vivo. The study employed a *Staphylococcus aureus (S. aureus)* biofilm-infected femur defect model in Sprague–Dawley (SD) rats (Fig. 7a). To evaluate the photothermal performance of the implants, localized NIR-II laser irradiation was applied for 15 min. Remarkably, the temperature at the Ti/ePt-Au NBPs/RGDC implant site surged from 32 °C to 50 °C, whereas the Ti control group exhibited only a modest increase from 32 °C to 38 °C (Fig. 7b, c). It is worth noting that the temperature elevation to 50 °C remains within the therapeutic window for biofilm eradication without inducing thermal necrosis in surrounding tissues, as supported by prior studies^49,50^. This pronounced photothermal response underscores the plasmonic enhancement of the ePt-Au NBPs architecture, which efficiently converts NIR-II light into localized hyperthermia, a critical feature for biofilm disruption.

Next, 7 days post-implantation, the retrieved Ti/ePt-Au NBPs/RGDC implants were incubated in Luria-Bertani (LB) medium for 24 h. Visual inspection revealed a clear culture medium in the Ti/ePt-Au NBPs/RGDC group, contrasting sharply with the turbidity observed in the Ti control group (Fig. 7d). Quantitative plate counting assays also showed a significant reduction in bacterial colonization on Ti/ePt-Au NBPs/RGDC implants (Fig. 7e), further demonstrating the robust biofilm eradication capability of Ti/ePt-Au NBPs/RGDC. These results are attributed to the synergistic effects of photothermal ablation and POD-like catalytic activity, where nanozyme-mediated •OH disrupted biofilm integrity while localized heating potentiated bacterial lethality.

Histopathological examination provided deeper insights into the anti-antibacterial infection mechanism. In clinical practice, when implants are infected, there is a rapid migration of inflammatory cells to the site of infection in response to the presence of pathogens. H&E staining of implant surrounding soft tissues in the Ti group revealed substantial neutrophil infiltration (Figs. 7f and S28a), indicative of a severe inflammatory response. Conversely, the Ti/ePt-Au NBPs/RGDC group exhibited minimal inflammation, manifesting its effective antibacterial efficacy. Complementary Giemsa staining (Figs. 7g and S28b) identified abundant bacterial clusters in the Ti group, whereas the Ti/ePt-Au NBPs/RGDC group showed negligible bacterial presence, which was consistent with the quantitative culture data. All those results demonstrated that the developed NIR-II-triggered plasmonic catalysis with tip-localized enhancement exemplifies a multifunctional platform for combating implant-associated infections. The synergistic ability of photothermal therapy with •OH generation of nanozyme under NIR-II illumination not only eradicated biofilms but also mitigated inflammatory responses, making it a promising candidate for clinical translation.

### In vivo osteogenic performance evaluation

**Fig. 8:**
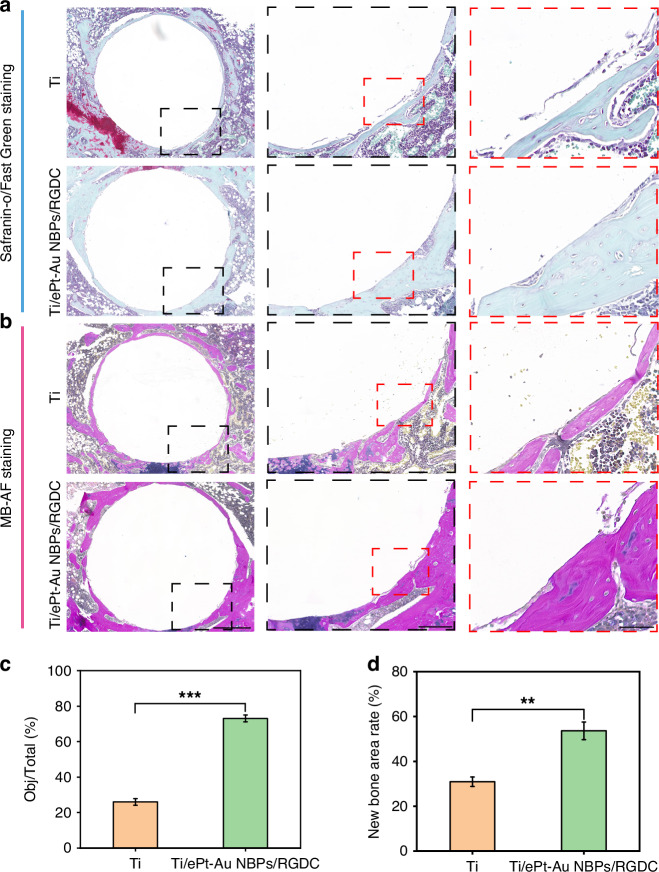
In vivo osteogenic performance evaluation of different implants. **a** Images of Safranin-O/Fast Green staining around the implants. **b** Images of Methylene blue-acid fuchsin staining near the implants. Scale bars: 500 μm (left), 200 μm (middle), and 50 μm (right). **c** Quantitative analysis of Safranin-O/Fast Green staining images. **d** Quantitative analysis of Methylene blue-acid fuchsin staining images. (*p < 0.05, **p < 0.01, and ***p < 0.001)

In addition to antibacterial performance, bone integration is also crucial for orthopedic implants. To assess bone integration, decalcified femoral tissues surrounding the implants were histologically analyzed using Safranin-O/Fast Green and Methylene blue-acid fuchsin staining (Fig. 8). Safranin-O/Fast Green staining revealed distinct osteogenic differentiation patterns, with mature bone matrices being stained green. Quantitative analysis demonstrated that Ti/ePt-Au NBPs/RGDC implants exhibited a significantly higher osteogenic differentiation index compared to the pure Ti group (Fig. 8a, c), which can be attributed to the RGDC-mediated enhancement of osteoblast adhesion and mechanotransduction. Furthermore, methylene blue-acid fuchsin staining highlighted newly formed bone (red staining) at the bone-implant interface, with Ti/ePt-Au NBPs/RGDC implants showing ~1.7-fold increase in new bone area relative to Ti (Fig. 8b, d). This accelerated osteogenesis may stem from the effects of the RGD peptide with the capacity to promote tissue regeneration.

Moreover, the biocompatibility of Ti/ePt-Au NBPs/RGDC implants was corroborated by histological evaluation of major organs (heart, liver, spleen, lung, and kidney) in SD rats. As displayed in Fig. S29, the major organs did not show pathological abnormalities after 28 days of implantation. Meanwhile, Ti/ePt-Au NBPs/RGDC implants showed favorable photothermal and photocatalytic stability after 28 days of implantation (Fig. S30). This observation disclosed the suitability of Ti/ePt-Au NBPs/RGDC implants for long-term biomedical applications.

## Discussion

In this study, we successfully developed a NIR-II-triggered plasmonic catalysis with tip-localized enhancement, ePt-Au NBPs, by integrating Pt NPs onto the terminal ends of Au NBPs. This architecture was further functionalized with RGDC peptides and immobilized on Ti substrates via the electrostatic surface self-assembly method, forming a multifunctional platform for hypoxic bacterial biofilm elimination and osteogenic differentiation in bone implants. The unique tip-selective Pt NPs modification not only induced a redshift of the LSPR absorption peak from the NIR-I to the NIR-II window but also substantially augmented photothermal conversion and photocatalytic activity. These enhancements are attributed to the synergistic interplay between Pt NPs-mediated hot electron generation and optimized charge transfer dynamics, which collectively amplify ROS production under NIR-II irradiation. Mechanistic investigations revealed that the NIR-II-triggered LSPR effect drives the generation of •OH and hyperthermia, both critical for biofilm disruption. The photothermal-photocatalytic synergy exacerbates lipid peroxidation, destabilizes bacterial membranes, induces cytoplasmic protein leakage, and depletes intracellular GSH, resulting in potent bactericidal activity. The •OH radicals effectively degrade eDNA within EPS, a structural pillar of biofilms, thereby compromising their integrity and facilitating the hyperthermia penetration for bacterial killing. In addition, the RGDC modification not only enhances biocompatibility but also promotes osteoblast adhesion and differentiation, addressing the dual challenges of infection control and osseointegration in orthopedic implants. The findings establish a foundational framework for developing next-generation smart coatings to combat implant-associated biofilm infections while fostering bone regeneration.

## Materials and methods

### Synthesis of Au NBPs

The synthesis of Au NBPs was carried out using the seed growth method. A mixture of chloroauric acid (HAuCl_4_, 0.01 M, 0.125 mL) and trisodium citrate (0.01 M, 0.25 mL) in 9.625 mL of deionized water served as the base solution. Subsequently, a freshly prepared frozen solution of sodium borohydride (NaBH_4_, 0.01 M, 0.15 mL) was added to the mixture under continuous stirring for 15 s. The resulting seed solution was stored at room temperature for 2 h. To create the reaction solution, cetyltrimethylammonium bromide (CTAB, 0.1 M, 40 mL) was mixed with HAuCl_4_ (0.01 M, 2 mL), silver nitrate (AgNO_3_, 0.01 M, 0.4 mL), hydrochloric acid (HCl, 1 M, 0.8 mL), and ascorbic acid (AA, 0.1 M, 0.32 mL). Then, 0.2 mL of the seed solution was introduced into the mixture while continuously stirring for 10 s. The reaction solution was then allowed to stand overnight at room temperature. Purification of the obtained Au NBPs was accomplished using BDAC deposition separation.

### Synthesis of ePt-Au NBPs

The purified 2 mL Au NBPs were subjected to centrifugation at a speed of 7000 rpm for 10 min. The resulting precipitate was dispersed in cetyltrimethylammonium chloride (CTAC, 0.08 M, 2 mL), and then AgNO_3_ (0.01 M, 10 μL) and AA (0.1 M, 5 μL) were added. These mixed solutions were placed in a temperature-controlled air shaker set at 60 °C and 100 rpm for 4.5 h. Subsequently, centrifugation was carried out at 6000 rpm for 10 min. The precipitate obtained from this process was redispersed in CTAB (0.003 M, 2 mL). Following this, chloroplatinic acid (H_2_PtCl_6_, 0.001 M, 50 μL) and AA (0.01 M, 50 μL) were introduced. The mixed solution was left undisturbed overnight at room temperature and subsequently subjected to centrifugation at 4000 rpm for 10 min. The resulting precipitate was then redispersed in 2 mL of water for storage purposes.

### Synthesis of Ti/ePt-Au NBPs/RGDC

Medical pure Ti plates and rods were initially polished using sandpaper of varying grit sizes to achieve a smooth surface. Subsequently, the polished Ti substrate underwent a sequential cleaning process involving ultrasonic treatment with acetone, anhydrous ethanol, and deionized water for 15 min. Solutions of Au NBPs and ePt-Au NBPs were prepared at a concentration of 5 mg mL^−1^. By the electrostatic surface self-assembly technique, positively charged 3-aminopropyl trimethoxysilane (APS) and negatively charged polystyrene sulfonate (PSS) were used as coupling agents to deposit Au NBPs and ePt-Au NBPs on the surface of the Ti substrate. The resulting Ti/ePt-Au NBPs samples were then immersed in RGDC peptide solutions and soaked at room temperature for 12 h. Finally, the Ti/ePt-Au NBPs/RGDC samples were obtained after removing the unreacted RGDC peptide solution through repeated washing with deionized water. Prior to performing any biological assays, all samples underwent sterilization using UV light.

### Bacterial and biofilm culture

*S. aureus* was obtained from ATCC (25923) and cultured in LB liquid medium. A bacterial suspension with a concentration of 1 × 10^6^ cfu mL^−1^ and a volume of 500 μL was applied to the surface of various samples placed in a 24-well plate. During the incubation period, the culture medium was replaced every 12 h to allow the growth of a bacterial biofilm on the sample surface. The different assay groups comprised: (1) Ti, (2) Ti + H_2_O_2_, (3) Ti/ePt-Au NBPs/RGDC, (4) Ti/ePt-Au NBPs/RGDC + H_2_O_2_, (5) NIR + Ti, (6) NIR + Ti + H_2_O_2_, (7) NIR + Ti/ePt-Au NBPs/RGDC, (8) NIR + Ti/ePt-Au NBPs/RGDC + H_2_O_2_, and (9) NIR + Ti/ePt-Au NBPs/RGDC + H_2_O_2_ + hypoxia. The NIR groups were continuously exposed to a fiber-optic system with a continuous-wave semiconductor laser (1064 nm, 0.6 W cm^−2^) for 15 min. Antibacterial and antibiofilm evaluations included plate coating assay, live/dead staining assay, SEM observation, and crystal violet staining.

### Cell culture and measurements

MC3T3-E1 cells were obtained from Pricella (CL-0378) and incubated in the complete growth medium (CM-0378, Procell). A suspension of MC3T3-E1 cells (5 × 10^5^ cells mL^−1^, 500 μL) was seeded onto distinct sample surfaces. Cell adhesion, cell viability, and osteogenic differentiation assays were carried out after culture for different times.

### Animals

The Sprague–Dawley (SD) rats (350 ± 50 g) were obtained from Guangxi Medical University Animal Center. All animal assays were performed with the prior approval of the First Affiliated Hospital of Guangxi Medical University (2024-E666-01). In vivo antibacterial, osteogenic, and biocompatibility assessment were performed either 7 or 28 days after implantation surgery.

### Implantation surgery

The Sprague–Dawley (SD) rats were segregated into two distinct groups: NIR + Ti and NIR + Ti/ePt-Au NBPs/RGDC. To construct biofilm on already-formed implants, Ti or Ti/ePt-Au NBPs/RGDC were incubated with bacteria for 48 h. Before surgery, SD rats were anesthetized via intraperitoneal injection of pentobarbital. Afterward, a surgical operation was performed in a sterile manner. A 5 mm skin incision was made at the femur near the knee joint, and the flat lateral surface of the femur was selected as the surgical site. A narrow channel with a diameter of about 1.2 mm was created at the femur using a 1 mm surgical drill. Subsequently, different implant rods coated with biofilm were gently implanted into the 1.2 mm channel. Then, the soft tissue and skin were carefully sutured. After implantation for one day, the implant sites were exposed to a fiber-optic system with a continuous-wave semiconductor laser (1064 nm, 0.6 W cm^−2^) for 15 min and the corresponding temperatures were recorded. In the rat bone defect model, we typically observe significant biofilm removal and new bone formation in the defect area by 7 and 28 days, when a clear, quantifiable assessment of biofilm eradication and bone regeneration can be observed. Subsequently, euthanasia was carried out after 7 and 28 days via pentobarbital administration.

## Supplementary information

NIR-II-triggered plasmonic catalysis with tip-localized enhancement: a strategy for hypoxic biofilm eradication on orthopedic implants

## Data Availability

The data presented in the manuscript is available from the corresponding author upon reasonable request.
