# Supplementary material for: NIR-II-triggered plasmonic catalysis with tip-localized enhancement: a strategy for hypoxic biofilm eradication on orthopedic implants
Source: Light Sci Appl. 2026 Apr 17;15:204. doi: 10.1038/s41377-026-02279-5 (PMC13090398; doi:10.1038/s41377-026-02279-5)
Supplement: Supplementary file 1 — NIR-II-triggered plasmonic catalysis with tip-localized enhancement: a strategy for hypoxic biofilm eradication on orthopedic implants [file 41377_2026_2279_MOESM1_ESM.docx]

**Supplementary Information for**

**NIR****-II****-****triggered** **plasmonic** **catalysis** **with tip-localized enhancement: a strategy for hypoxic biofilm eradication on orthopedic implants**

Yu Sun ^a^, Fanglin Sheng ^b^, Yi Liang ^b^, Jinhui Meng ^b^, Ke Huang ^b^, Sanmao Liu ^a^, Yingfeng Qin ^b,*^, Maolin He ^a^^,*^, Jin-Wen Liu ^b,*^

^a^ *Division of Spinal Surgery, The First Affiliated Hospital of Guangxi Medical University, Nanning 530021, China*

^b^ *Key Laboratory of Longevity and Aging-related Diseases of Chinese Ministry of Education,* *Guangxi Colleges and Universities Key Laboratory of Biological Molecular Medicine Research, School of Basic Medical Sciences Guangxi Medical University, Nanning 530021, China*

^*^ Corresponding authors.

E-mail address: qinyingfeng@sr.gxmu.edu.cn (Y. Qin), hemaolin@stu.gxmu.edu.cn (M. He), jinwenliu@hnu.edu.cn (J-W. Liu).

**Materials**

Chloroauric acid (HAuCl_4_), trisodium citrate, cetyltrimethylammonium bromide (CTAB), cetyltrimethylammonium chloride (CTAC), sodium borohydride (NaBH_4_), silver nitrate (AgNO_3_), hydrochloric acid (HCl), ascorbic acid (AA), chloroplatinic acid (H_2_PtCl_6_), benzyldimethylhexadecylammonium chloride (BDAC), 3-amino-propyl trimethoxysilane (APS), polystyrene sulfonate (PSS), 3,3′,5,5′-tetramethylbenzidine hydrochloride dehydrate (TMB), hydrogen peroxide (H₂O₂), terephthalic acid (TA), 5, 5-dimethyl N-oxide pyrroline (DMPO), luria-bertani (LB) broth and crystal violet were purchased from Aladdin Industrial Co. (China). Live/dead Baclight bacterial viability kit was obtained from Thermo Scientific (Shanghai, China). The RGDC peptide was purchased from Top-peptide Co. (Shanghai, China). Lipid peroxidation assay kit, 2-Nitrophenyl β-D-galactopyranoside (ONPG), reactive oxygen species (ROS) assay kit, 4',6-diamidino-2-phenylindole (DAPI), rhodamine-phalloidin, cell counting kit-8 (CCK-8) assay kit, bicinchoninic acid (BCA) assay kit, alkaline phosphatase (ALP) assay kit and osteogenesis assay kit were obtained from Beyotime Biotechnology Co. (Jiangsu, China). Medical pure Ti plates (6 mm in diameter, 2 mm in thickness) and rods (2 mm in diameter, 6 mm in thickness) were purchased from Fu-Tai Metal Materials Co. (China). Milli-Q water (18 MΩ cm) was applied for all solution preparations. In vitro and in vivo experiments, we used a continuous-wave (CW) fiber-coupled semiconductor laser (model: PL-LED100F, Princess, Beijing, China) as the second near-infrared (NIR-II) light source, with an output wavelength of 1064 nm and a power of 0.6 W cm^-2^.

**Characterization**

The morphology of nanoparticles was analyzed using a transmission electron microscope (TEM) (JEM 2100, JEOL, Tokyo, Japan). The UV-Vis-NIR absorbance spectrum was recorded with a UV-3600 spectrophotometer (Shimadu, Kyoto, Japan). The crystalline phase structure was investigated using X-ray diffraction (XRD) (D8A25, Bruker, Germany). Zeta potential measurements were conducted using a Zetasizer Nano ZS system (Malvern, UK). The surface morphology of implants was characterized by field-emission scanning electron microscopy (FE-SEM) (JSM-7800F, JEOL, Japan). The surface chemical composition of implants was obtained through X-ray photoelectron spectroscopy (XPS) (ESCALAB 250Xi, Thermo, Waltham, USA). To determine the wettability of different implant samples, water contact angle measurements were performed (Model 200, Future Scientific, Taiwan, China). For photocatalytic and photothermal reaction systems, NIR-II irradiation (1064 nm, 0.6 W cm^-2^) was performed through a fiber-optic system with a continuous-wave semiconductor laser (PL-LED100F, Princess, China). Photothermal imaging was conducted using an FLIR E50 instrument (FLIR Systems, Inc, USA).

**Measurement of photothermal performance and peroxidase-like activity of nanoparticles**

In a 24-well plate, solutions of Au NBPs or ePt-Au NBPs (160 μg mL^-1^, 500 μL) were added and exposed to a fiber-optic system with a continuous-wave semiconductor laser (1064 nm, 0.6 W cm^-2^). The photothermal performance of the nanoparticles was studied by monitoring the temperature variation over time. To evaluate the peroxidase-like activity of different nanoparticles in the presence of H_2_O_2_, a TMB substrate solution was prepared using acetic acid-sodium acetate (HAc-NaAc) buffer solution (0.1 M, pH 5.5). At room temperature, a mixture of 160 μg mL^-1^ nanoparticles, 0.3 mM TMB solution, and 0.2 mM H_2_O_2_ solution was added to a 3 mL HAc-NaAc buffer solution. After a reaction time of 10 min under NIR-II irradiation or in the dark, the absorbance of the reaction system at 652 nm was measured using UV-Vis-NIR spectroscopy.

For kinetic analysis, a constant concentration of 160 μg mL^-1^ ePt-Au NBPs in a 3 mL HAc-NaAc buffer solution was maintained along with a fixed concentration of 0.2 mM H_2_O_2_. The TMB concentration was varied from 0.1 to 1.6 mM, or the TMB concentration was kept at 0.3 mM while the H_2_O_2_ concentration was varied from 0.0625 to 1 mM under NIR-II irradiation. A control experiment without NIR-II irradiation was also conducted. The Michaelis-Menten constant (*K*m) was calculated using the formula: 1/*V* = *K*m/*V*max (1/[*S*] + 1/*K*m), where *V* represents the initial velocity, *V*max is the maximum reaction rate, [*S*] is the substrate concentration, and *K*m corresponds to the Michaelis constant.

Additionally, a cyclic experiment was performed on the ePt-Au NBPs solution under NIR-II irradiation. After five cycles of continuous irradiation and cooling, temperature, catalytic activity, UV absorption, and morphology of the ePt-Au NBPs were observed.

**FDTD simulations**

For the computational simulation, the finite-difference-time-domain (FDTD) method with perfectly matched layer (PML) boundary conditions was employed. This simulation was performed using the FDTD solutions software developed by Lumerical in Canada. A total-field scattered-field (TFSF) source with a wavelength of 1064 nm was used in simulation. The incident wave propagated along the negative z-axis, and the electric field was polarized parallel to the long axis of the nanoparticles (x-axis). The optical constants of Au used in the simulation were based on the values measured by Johnson and Christy. The geometric dimensions of the Au NBPs were selected to match the average values obtained experimentally. In the case of the ePt-Au NBPs model, Pt nanoparticles were positioned at both ends of the Au NBPs to simulate the hybrid structure. The refractive index of the surrounding medium was set to 1.33. A uniform mesh size of 0.5 nm was employed throughout the simulation region to ensure sufficient spatial resolution, particularly at the sharp tip regions and Pt nanoparticle clusters. The electric field distribution was monitored in the x-y plane using a frequency-domain field monitor. To evaluate the near-field enhancement, the field enhancement factor was defined as |*E*|^2^/|*E*_0_|^2^, where *E* represents the local complex electric field amplitude and *E*_0_ denotes the amplitude of the incident electric field. The maximum field enhancement factors at the tip regions were extracted for quantitative comparison.

**Electrochemical characterization of NPs**

Electrochemical characterization was carried out using an electrochemical workstation, which consisted of a glassy carbon electrode (GCE) as the working electrode, a Pt counter electrode, and an Ag/AgCl reference electrode. Solutions of Au NBPs and ePt-Au NBPs were drop-casted onto a clean electrode with an area of 0.07 cm² to prepare the GCE working electrode. Under NIR-II irradiation, the photocurrent density was measured in a Na_2_SO_4_ electrolyte solution with a concentration of 0.5 M at a bias potential of 0.4 V. Electrochemical impedance spectroscopy (EIS) was conducted in a 0.1 M Na_2_SO_4_ electrolyte solution over a frequency range of 0.01 to 10^5^ Hz at open-circuit potential.

**NIR-II-enhanced peroxidase-like activity of Ti/ePt-Au NBPs/RGDC**

To evaluate the photothermal performance of different implants, they were placed in a 24-well culture plate, and 1 mL of PBS solution was added. The samples were continuously exposed to a fiber-optic system with a continuous-wave semiconductor laser (1064 nm, 0.6 W cm^-2^) for 15 min. The temperature increase curves and thermal imaging photos were used to assess the photothermal effect of the implants. For peroxidase-like activity assessment, TMB solution (0.02 M, 50 μL), H_2_O_2_ solution (0.1 M, 50 μL), and 0.9 mL of NaAc-HAc buffer solution were added to the surface of the different implants. After a reaction time of 15 min, the absorbance of the reaction solution at 652 nm was measured using UV-Vis-NIR spectroscopy. Further investigations were conducted by adjusting the temperature, pH value, and light intensity to explore the peroxidase-like catalytic activity of the Ti/ePt-Au NBPs/RGDC samples. Additionally, a cyclic experiment was performed by subjecting the Ti/ePt-Au NBPs/RGDC samples to NIR-II irradiation. After five cycles of continuous irradiation and cooling, the temperature and catalytic activity of the ePt-Au NBPs were observed.

**Detection of •OH**

The fluorescence probe TA was used to assess the production of ·OH and form a fluorescent product, 2-hydroxyterephthalic acid (TAOH). Specifically, TA (0.005 M, 500 μL) and H_2_O_2_ (0.01 mM, 500 μL) were added to the Ti/ePt-Au NBPs/RGDC sample and mixed well. The mixture was allowed to react for 15 min with or without NIR-II irradiation. After incubating the reaction system at room temperature for 12 h, the fluorescence intensity was measured. The production of ·OH was further determined using electron spin resonance (ESR) spectroscopy, with DMPO serving as a free radical scavenger. The assay was conducted in 1 mL of NaAc-HAc buffer solution with an H_2_O_2_ concentration of 0.5 mM and a DMPO concentration of 0.2 mM. The mixture was allowed to react for 15 min with or without NIR-II irradiation and was aspirated into a quartz capillary tube for ESR analysis.

**Plate coating assay**

After different treatments, the bacterial liquid was uniformly dispersed, and a 20 μL sample of the bacterial liquid was extracted. Subsequently, this sample was diluted using LB liquid medium in a 1:100 ratio. Then, 100 μL of the diluted sample was evenly spread onto an LB solid medium plate, which was subsequently incubated at 37 ℃ for 24 h. Following the completion of the incubation period, the number of colonies formed on the plate was enumerated.

**Live/dead staining assay**

Bacterial viability on different samples was detected using a live/dead BacLight bacterial viability kit. STYO9 and PI dyes were employed to stain the bacteria or biofilm in the dark for 30 min. The excess dye in the reaction system was washed away with sterile PBS, and the samples were observed and photographed under a confocal laser scanning microscope (CLSM).

**Morphological observation**

The bacteria obtained from different groups were centrifuged and fixed with formaldehyde solution at 4 ℃ for 30 min. Gradient ethanol (20%, 40%, 60%, 80%, and 100%) was used for dehydration for 15 min each, followed by dehydration with tert-butanol for 30 min. Finally, SEM was utilized to evaluate the morphological changes of bacteria in different groups.

**Crystal violet staining**

A 1mL crystal violet solution (0.2%) was used to stain the biofilm. After incubation at room temperature for 30 min, the excess crystal violet was removed by washing three times with sterile PBS, and then the stained biofilm was photographed and observed. After photography, 1mL of 100% ethanol was added to dissolve the crystal violet dye, and the remaining amount of the biofilm was evaluated by measuring the absorbance of the resulting solution at 590 nm.

**Intracellular ROS test**

To detect intracellular ROS in bacteria, 2’,7’-dichlorodihydrofluorescein diacetate (DCFH-DA) fluorescence probe was employed. Bacteria treated with different groups were stained with DCFH-DA (20 μM) dye in the dark for 30 min. Then, excess dye was removed by washing the bacteria with sterile PBS buffer three times. Finally, the stained bacteria were observed under a CLSM microscope to determine the level of ROS in each group.

**Lipid peroxidation test**

Malondialdehyde (MDA) content is an important parameter for evaluating the body potential antioxidant capacity, representing the rate and intensity of lipid peroxidation and indirectly reflecting the degree of tissue oxidative damage. Bacteria treated with different groups were lysed with lysozyme and proteinase K. After centrifugation at 10000 rpm for 10 min, the supernatant was collected, and MDA was determined using lipid peroxidation assay kit.

**Membrane permeability test**

ONPG was used as a substrate to evaluate changes in bacterial membrane permeability. The more severe the bacterial membrane damage, the easier ONPG can penetrate into the bacteria and react with β-galactosidase (β-Gal) inside the bacteria to produce yellow o-nitrophenol, which has a strong absorption peak at 405 nm. ONPG solution (0.75 M, 500 μL) was added to bacteria treated with different groups, and then the reaction was carried out at room temperature for 30 min. The absorbance at 405 nm was recorded.

**Protein leakage test**

Bacteria treated with different groups were centrifuged at 12000 rpm for 5 min at 4 °C. The protein concentration in the supernatant was detected using a BCA assay kit, and the total protein mass in each group of bacteria was measured. Then, the protein leakage rate of each group was obtained by normalizing the protein concentration.

**GSH oxidation level test**

Glutathione (GSH) is a major endogenous antioxidant produced by cells that can be transformed into glutathione disulfide (GSSG) after oxidation. It can effectively prevent cell damage caused by oxidative stress. Ellman's reagent was used to determine the level of GSH oxidation. In different assay groups, 200 μL (0.8 mM) GSH carbonate buffer solution (50 mM, pH 8.7) was added to each well, and the reaction was carried out for 30 min. Afterward, the samples were treated for 15 min with or without NIR-II irradiation. After removing the samples, 450 μL (50 mM) Tris-HCl (pH 8.0) solution and 100 μL (10 mM) DTNB carbonate buffer solution (50 mM, pH 8.7) were added to each well. After thoroughly mixing on a shaker for 30 min, the absorbance at 410 nm was measured.

**Extracellular DNA (eDNA) degradation test**

The biofilm was gently scraped along the bottom and sidewalls of the 24-well plate using a cell scraper. eDNA was extracted from the biofilm using an enzyme extraction method. The extracted eDNA was tested in gel electrophoresis. 1,3-Dichloro-7-hydroxy-9,9-dimethyl-2 (9H)-acridone (DDAO) can bind to eDNA to produce red fluorescence for eDNA visualization. In the dark condition, DDAO dye solution was applied to the surface of the biofilm for 15 min. After slowly washing the remaining dye with sterile PBS buffer, the samples were observed and photographed under a CLSM microscope.

**Cell adhesion assay**

A suspension of MC3T3-E1 cells (5 × 10^5^ cells mL^-1^, 500 μL) was seeded onto distinct sample surfaces. Following incubation for 4 or 24 h, the cell culture medium was eliminated. The adhered MC3T3-E1 cells were then fixed with 4% paraformaldehyde at 4 °C for 30 min. Subsequently, treatment with 0.2% Triton X-100 for 2 min facilitated the staining of cytoskeleton actin by phalloidin for 30 min. Next, the cells in each well were treated with 200 μL DAPI staining reagent and incubated at 37 °C for 5 min. Using CLSM, we evaluated the morphology of MC3T3-E1 cells adhering to different samples. Further, ImageJ software was utilized to count the number of adherent cells and calculate the total spread area.

**Cell viability assay**

The CCK-8 assay kit was employed to determine cell viability of distinct samples. A suspension of MC3T3-E1 cells (5 × 10^5^ cells mL^-1^, 500 μL) was seeded on various sample surfaces and incubated for 1, 3, and 7 days. Next, in each well, 220 μL of CCK-8 working solution was supplemented, and the plate was incubated at 37 ℃ for 1 h. Thereafter, 200 μL of the mixture was transferred to a 96-well plate, and the absorbance was measured at 450 nm. To assess cell viability under NIR-II irradiation, the MC3T3-E1 cells on different sample surfaces were exposed to NIR-II laser for 15 min per day. Cell viability was evaluated after 1, 3, and 7 days of incubation.

**ALP activity**

For the evaluation of osteogenic differentiation and gene expression by monitoring ALP activity, the time (3 days, 7 days, 14 days and 28 days) were strategically chosen since they represent critical time points for investigating early stage of biofilm formation, early, mid-to-late stage osteogenic differentiation and significant new bone formation *in vitro*, respectively. In brief, MC3T3-E1 (500 μL, 5 × 10^5^ cells mL^-1^) suspension was inoculated into different samples. After cell culture for different days, the adherent MC3T3-E1 cells were washed three times with sterile PBS buffer. 1% Triton X-100 was added to each well, and the cells were lysed at 4 °C for 30 min. The ALP activity and total protein content were determined using ALP assay kit and BCA assay kit.

**Collagen secretion and** **matrix mineralization assay**

After cell culture for different days, the adherent MC3T3-E1 cells were washed three times with sterile PBS buffer. The MC3T3-E1 cells attached to the sample surface were fixed with 4% paraformaldehyde at 4°C for 30 min. The fixed MC3T3-E1 cells were stained with sirius red staining solution for 1h. NaOH solution (0.1 M, 200 μL) was added to dissolve the crystals in the stained cells. The dissolved mixture was transferred to a 96-well plate, and the secretion of collagen was quantified by measuring the absorbance at 540 nm. In addition, for matrix mineralization assay, the fixed cells were stained with 200 μL alizarin red solution (40 mM, pH 4.1). Then, 10% acetic acid was added and gently shaken. The shaken mixture was transferred to an EP tube and incubated in a water bath at 85 °C for 10min. Thereafter, 200 μL of 10% ammonia solution was then added, and the mineralization level of MC3T3-E1 in different samples was assessed by measuring the absorbance at 405 nm.

**Adhesion and osteogenesis related genes test**

After incubating for different times, the total RNA of the MC3T3-E1 cells was extracted using Trizol and RNA extraction reagents. cDNA products were obtained using a reverse transcription kit. *Integrin αv*, *Integrin β3*, *ALP*, *runt-related transcription factor 2 (Runx2)*, *type I collagen (Col I)*, and *osteocalcin (OCN)* primers were used to amplify genes, and *GAPDH* was used as an internal control for relative content comparison. Amplification conditions: 50 ℃, 120 s; 95 ℃, 120 s; followed by 95 ℃, 15 s and 60 ℃, 60 s, for a total of 40 cycles. Table S1 presents the sequences of the primers (Sangon, China) used in this study.

***In vivo* antibacterial activity**

Seven days after the operation, implants from both groups were removed and subjected to sonication using PBS. Subsequently, 50 μL of the resulting solution was extracted and cultured on LB solid medium and LB liquid medium. The cultures were then incubated at 37 °C for 24 h. The clone numbers and culture turbidity were recorded. Soft tissues surrounding the different implants were carefully collected and washed with sterile PBS buffer. Following this, the tissues were fixed in a 4% paraformaldehyde solution at 4 °C for 72 h. Dehydration was carried out using a gradient ethanol solution of 50%, 70%, 90%, and 100%. After permeating with xylene, the samples were routinely embedded in paraffin and 3-5 μm histological sections were prepared. Subsequently, sections were stained using hematoxylin-eosin (HE) and Giemsa.

***In vivo* osteogenic assessment**

The femur of both groups involved in the study were fixed using 4% paraformaldehyde solution at 4 °C for 72 h. Subsequently, the fixed bone tissue was decalcified by immersion in ethylenediaminetetraacetic acid (EDTA) solution, which was renewed every three days for four weeks in total. After the completion of the decalcification process, implants were cautiously removed from the fixed bone tissue utilizing a needle. Dehydration was carried out using a gradient ethanol solution consisting of 50%, 70%, 90%, and 100% over a period of 24 h. Following this, the dehydrated samples were permeated with xylene and embedded in paraffin. Bone tissue sections measuring approximately 5 μm in thickness were prepared. Sections were stained using Safranin-O/Fast Green and methylene blue-acid fuchsin.

***In vivo* biocompatibility assessment**

To evaluate the biosafety of different implants, major organs (heart, liver, spleen, lung, and kidney) were stained with HE after 4weeks of implantation surgery.

**Statistical Analysis**

The data were expressed as mean ± standard deviation of at least 3 samples (n ≥ 3). Statistical analysis was performed using Students’ t-test or one-way analysis of variance (ANOVA).


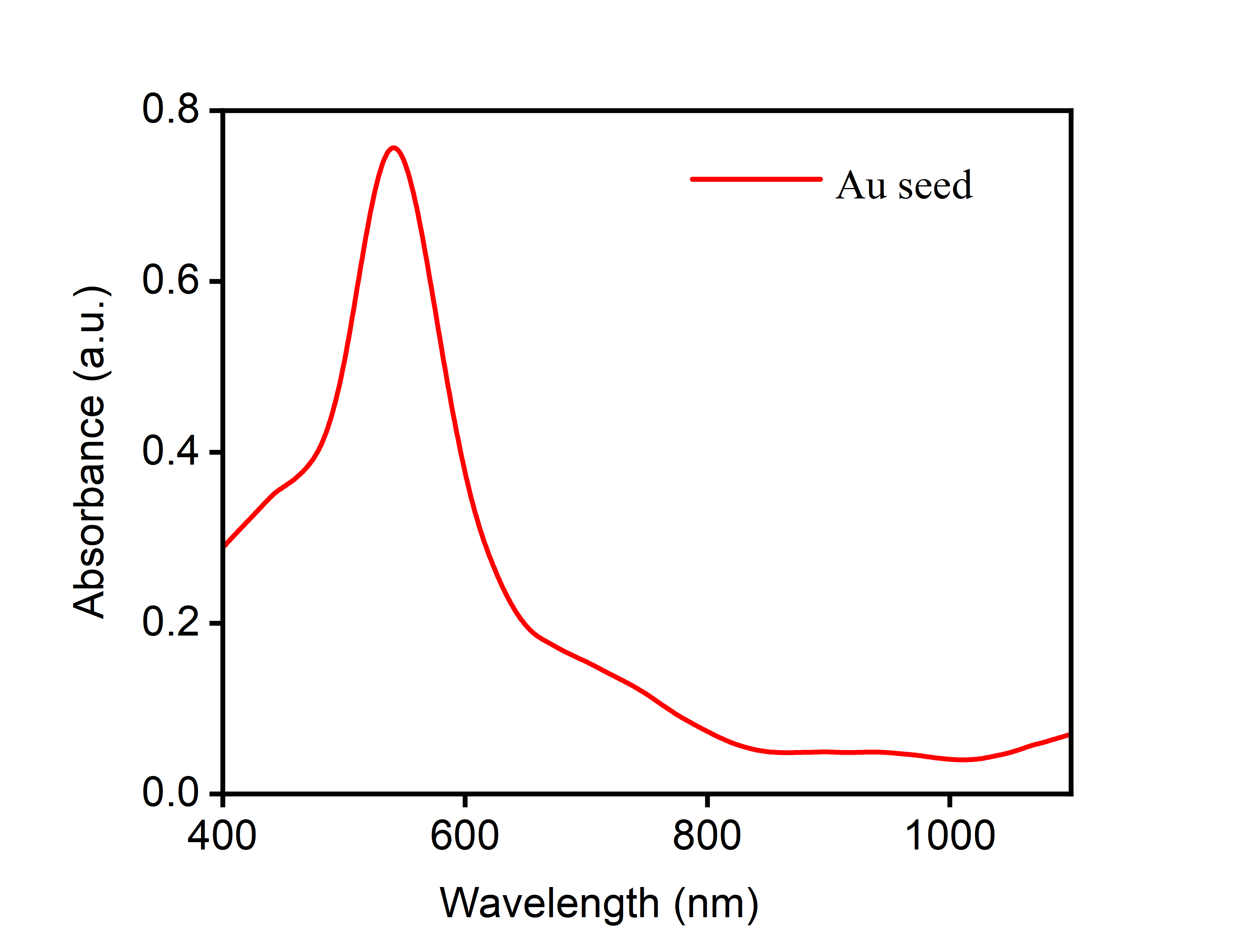


**Fig. S1** UV-Vis-NIR absorption spectra of Au seeds.


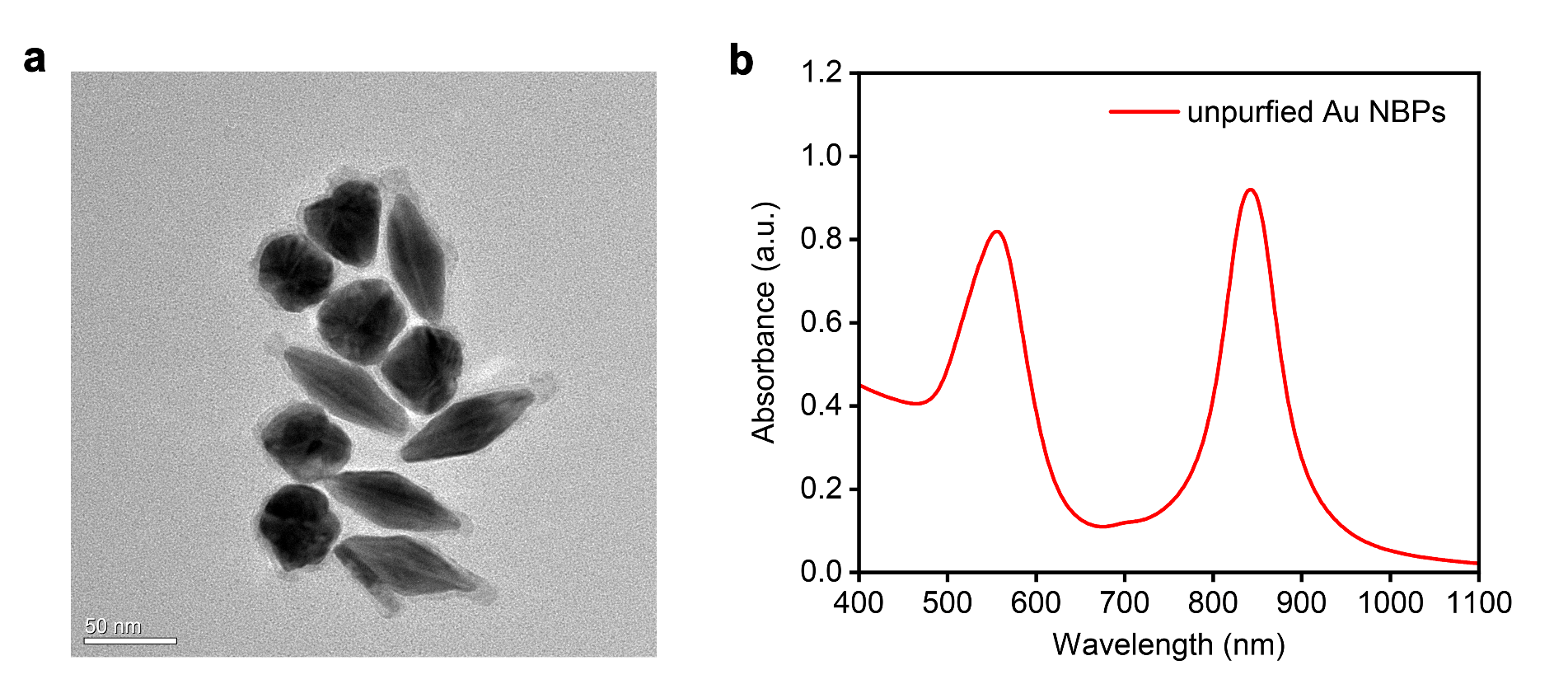


**Fig. S2** TEM image and UV-Vis-NIR spectrum of Au NBPs crude product. (a) TEM image. (b) UV-Vis-NIR spectrum.


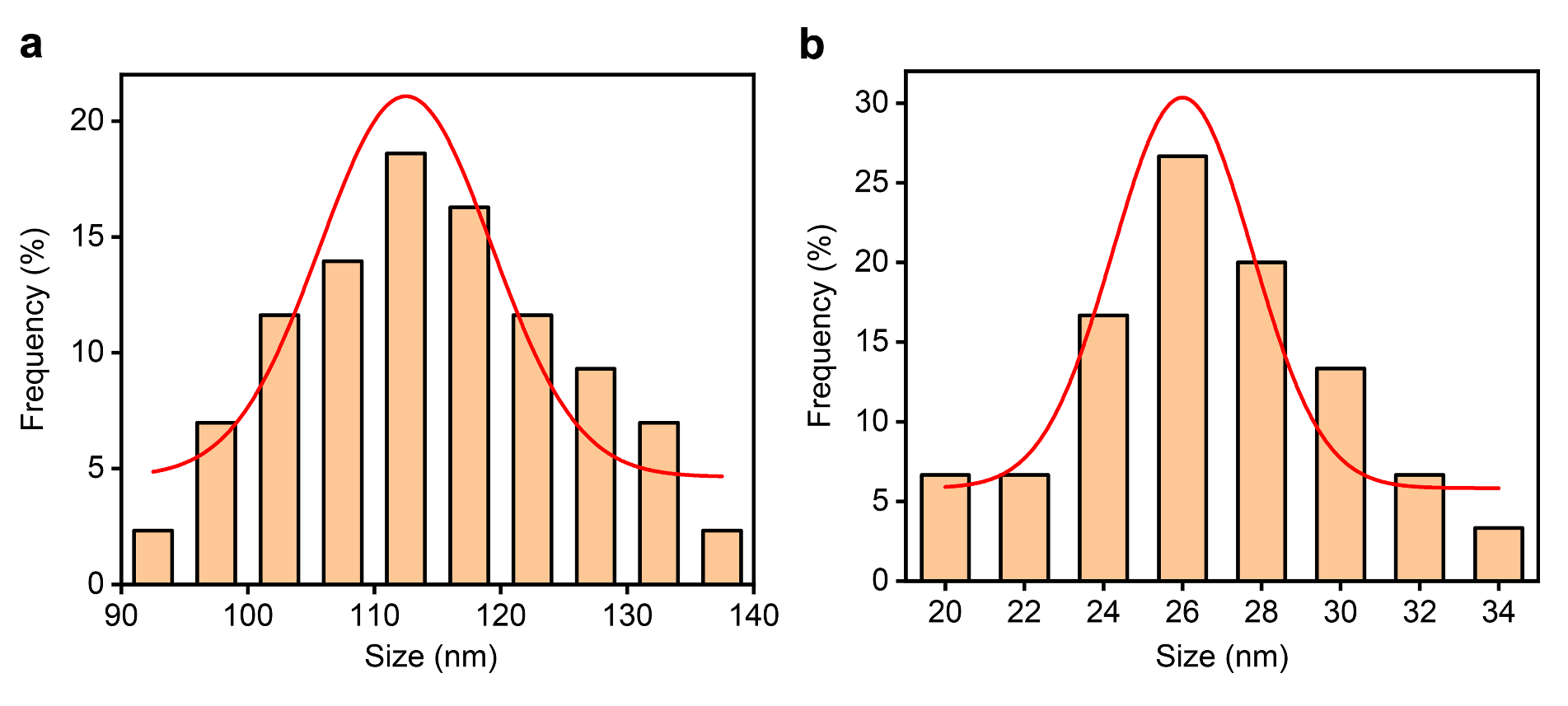


**Fig. S3** Particle size distribution of Au NBPs. (a) Length. (b) Diameter.


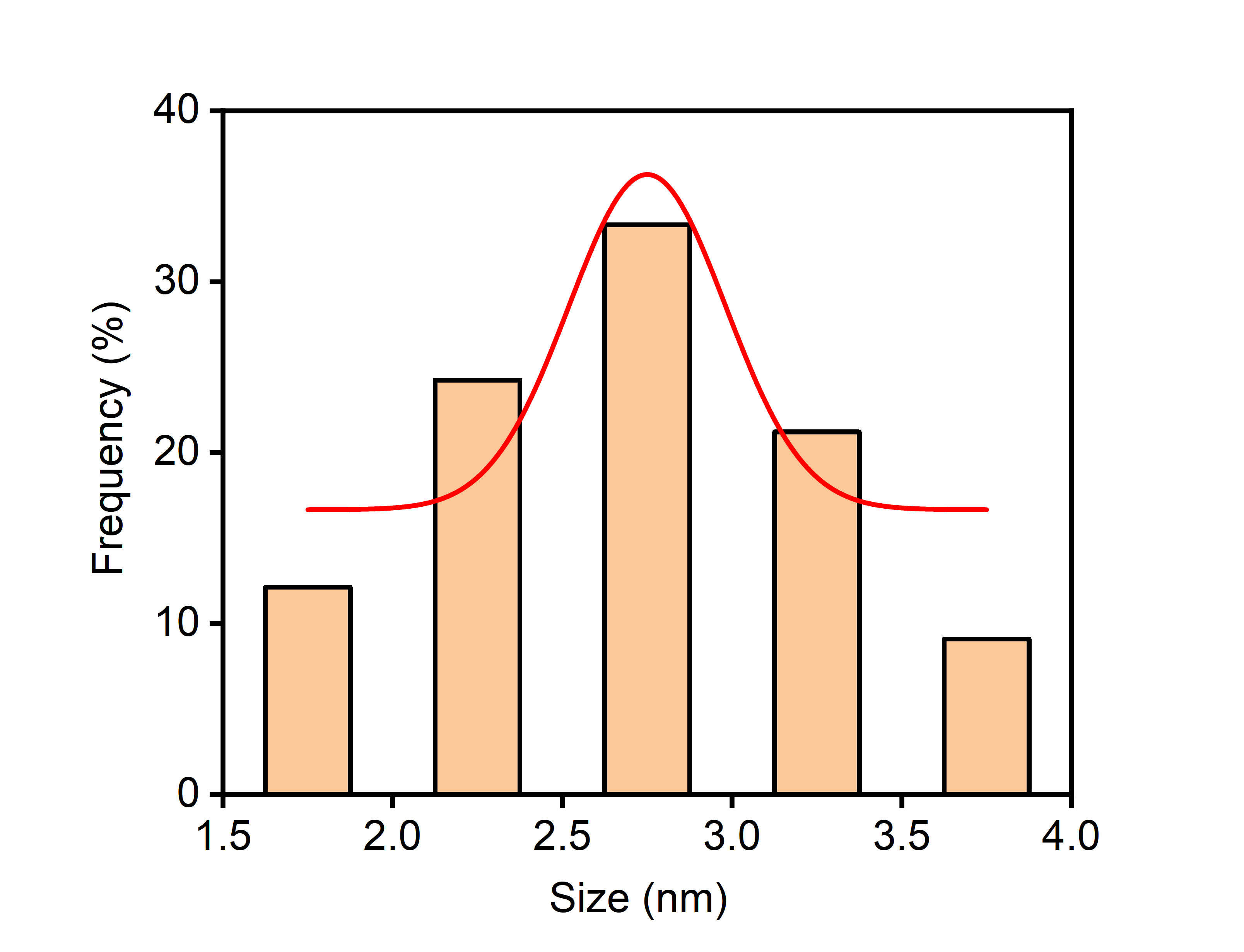


**Fig. S4** Particle size distribution of Pt NPs.


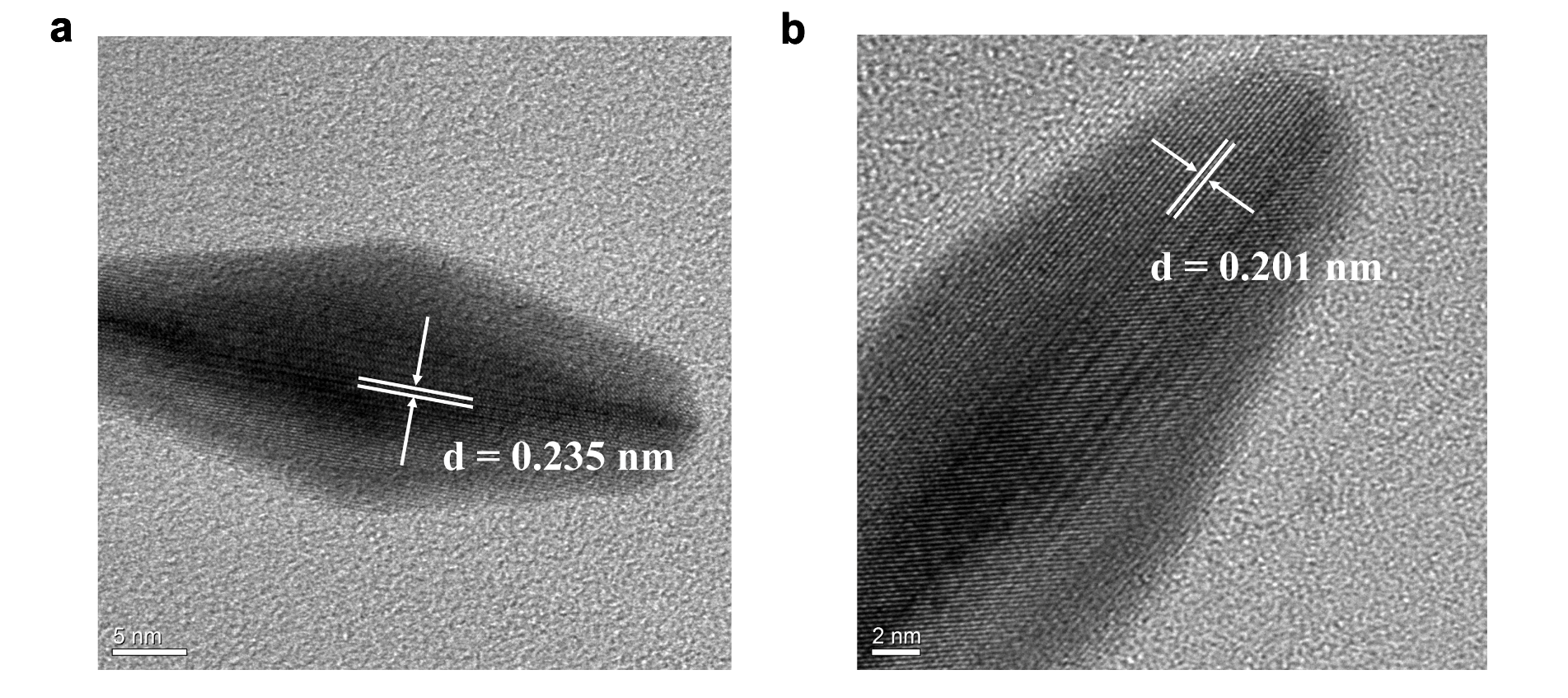


**Fig. S5** HRTEM image of Au NBPs.


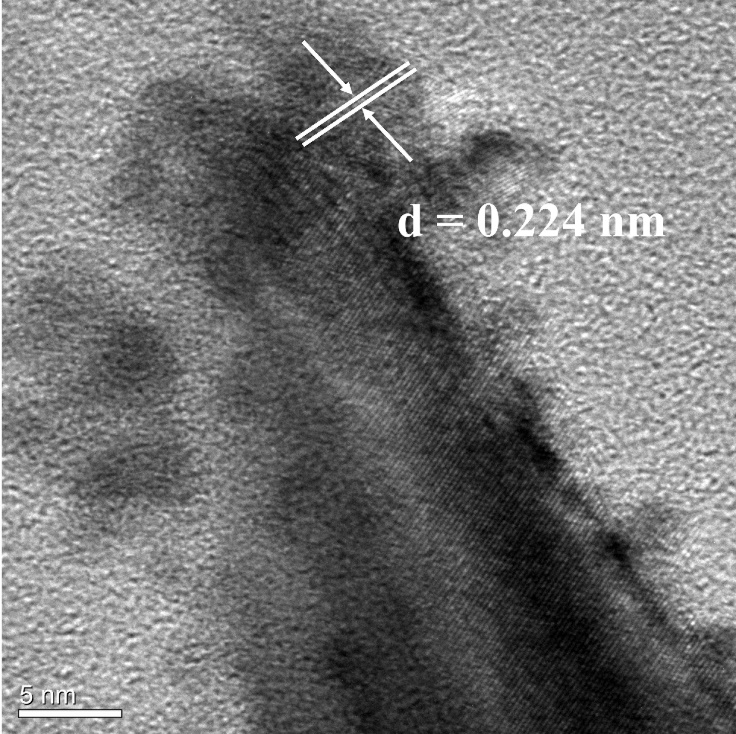


**Fig. S6** HRTEM image of ePt-Au NBPs.


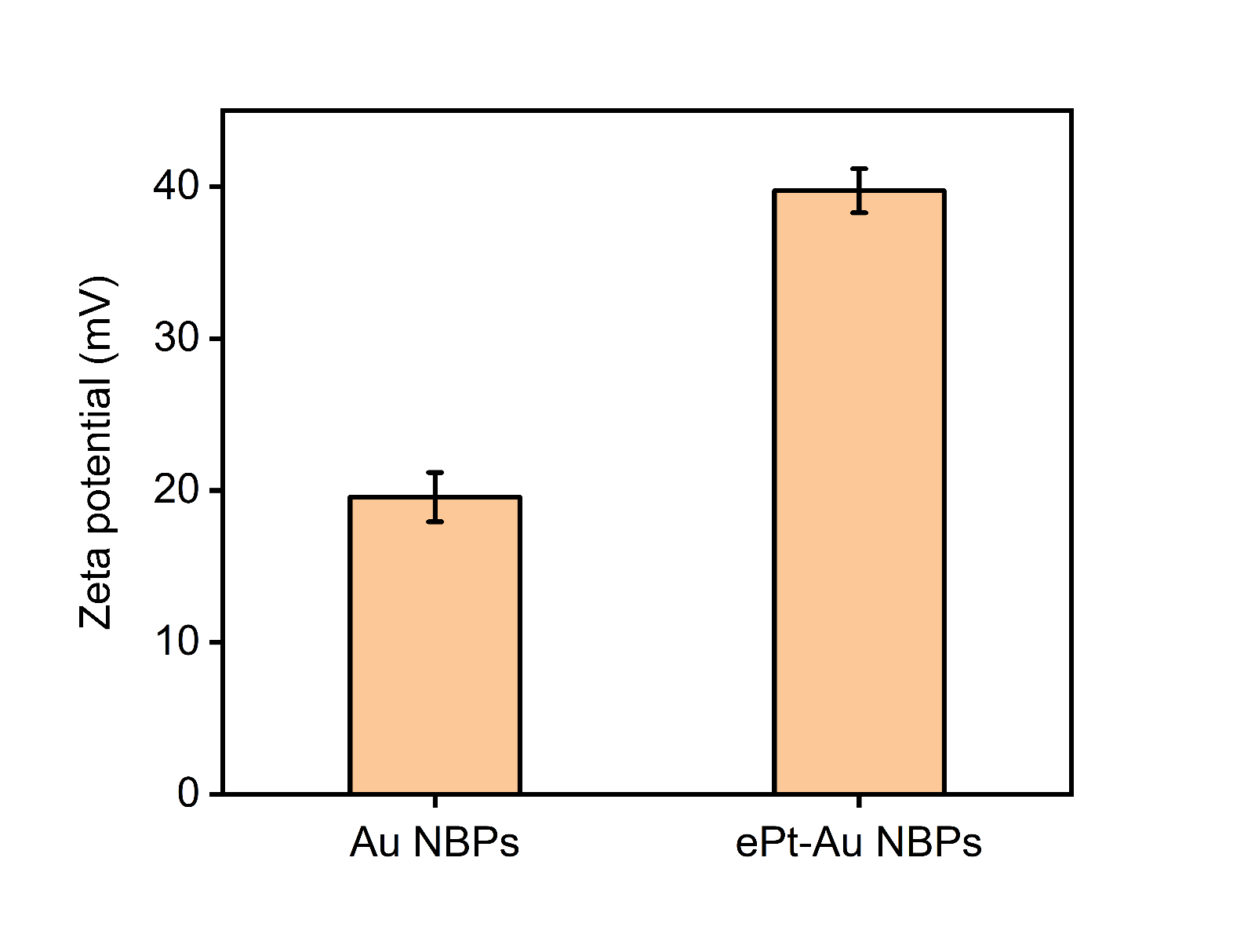


**Fig. S7** Zeta potential of Au NBPs and ePt-Au NBPs.


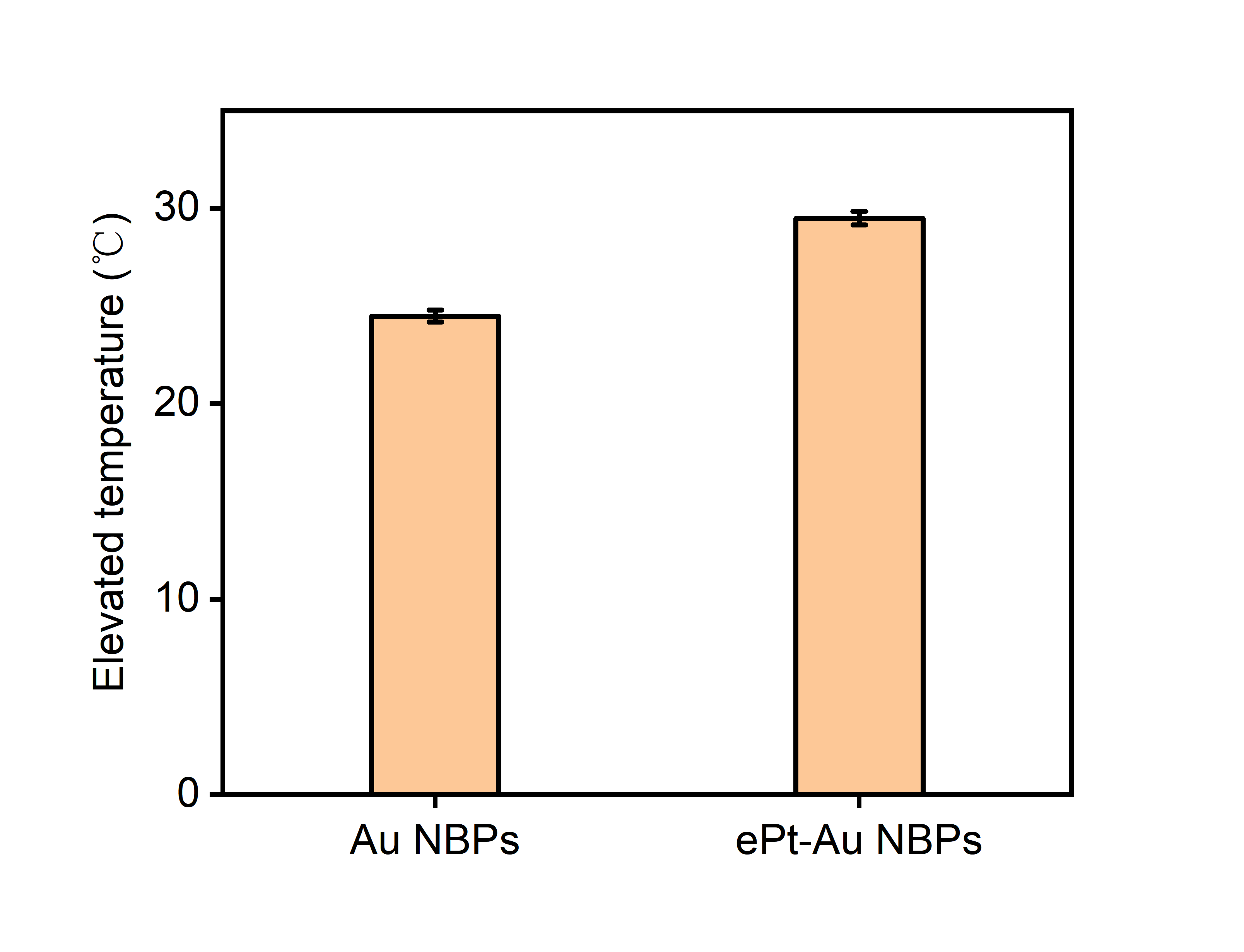


**Fig. S8** Relative temperature elevation of Au NBPs and ePt-Au NBPs (0.6 W cm^-2^).


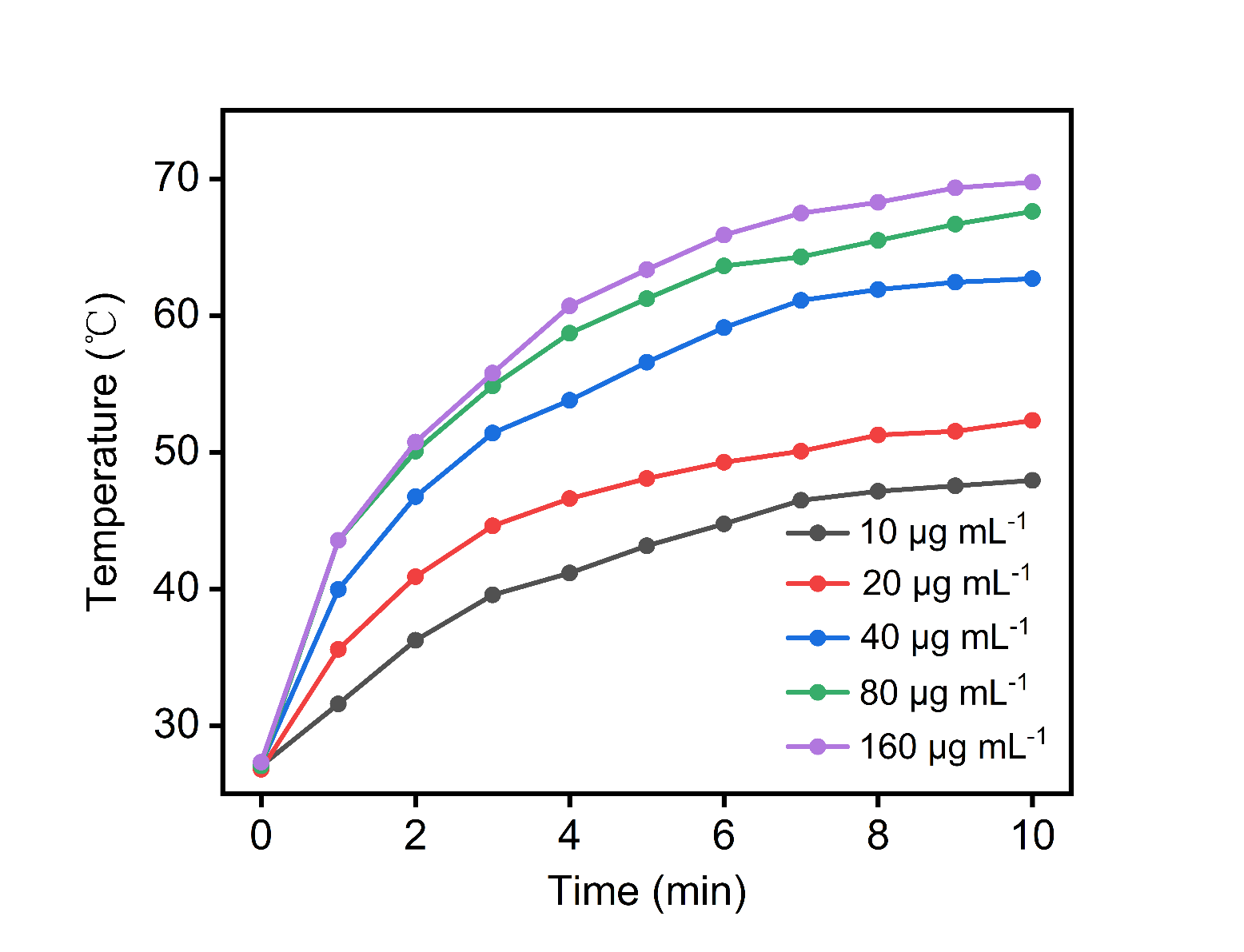


**Fig. S9** Temperature elevation curves of ePt-Au NBPs at different concentrations (0.6 W cm^-2^).


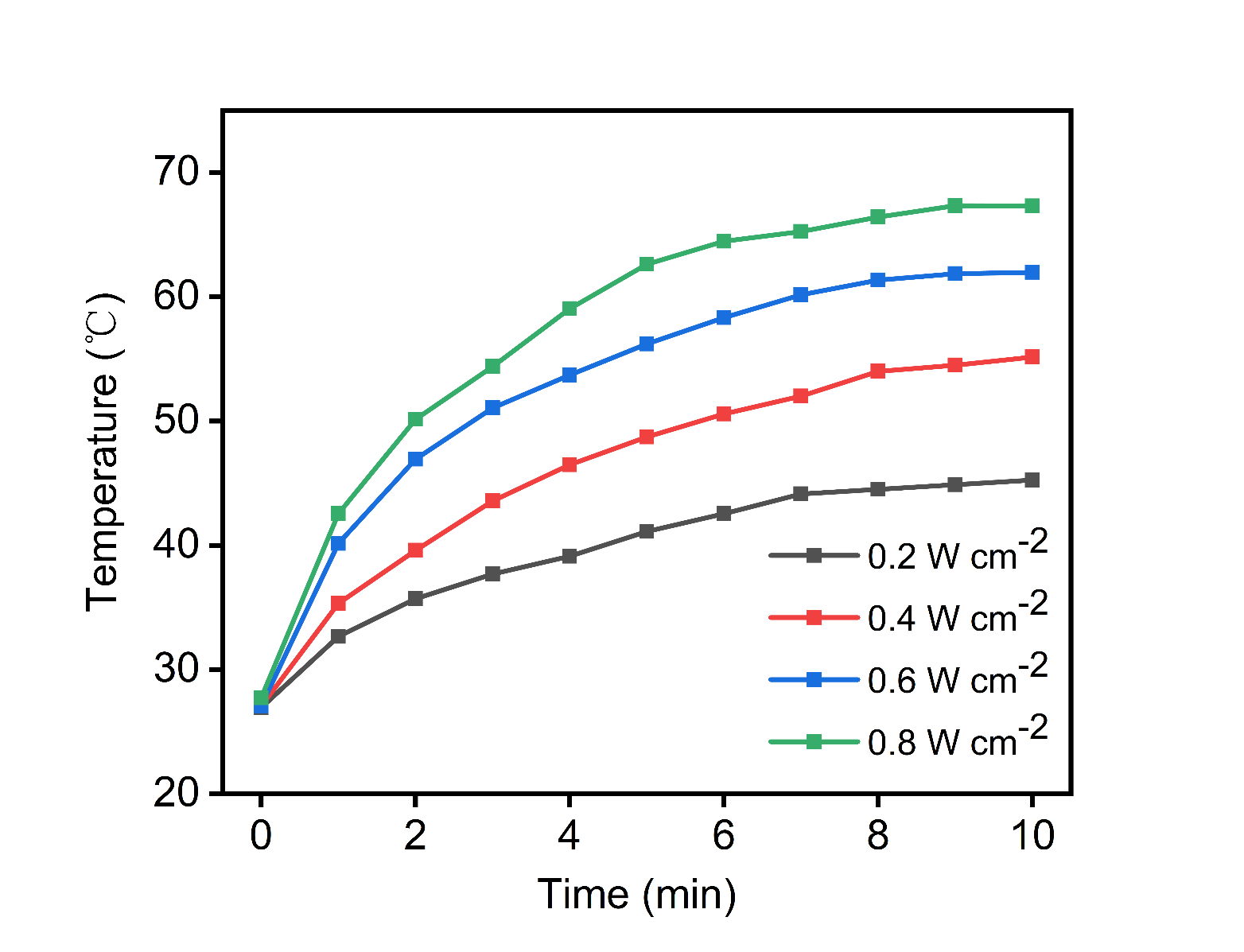


**Fig. S10** Temperature elevation curves of ePt-Au NBPs under different laser intensities.


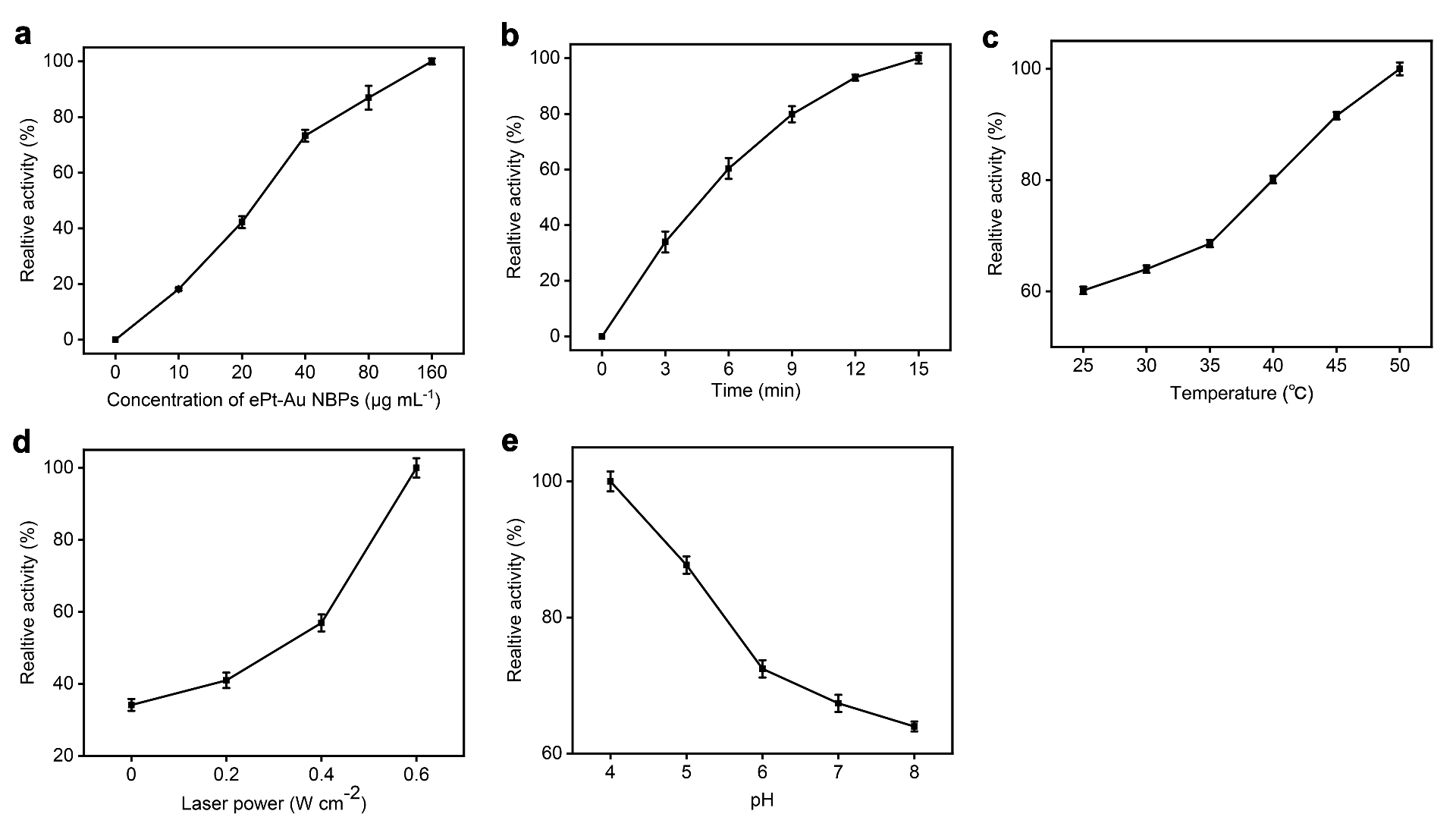


**Fig. S11** Factors influencing the peroxidase-like activity of ePt-Au NBPs. (a) Concentration of ePt-Au NBPs. (b) Time. (c) Temperature. (d) Laser intensity. (e) pH.


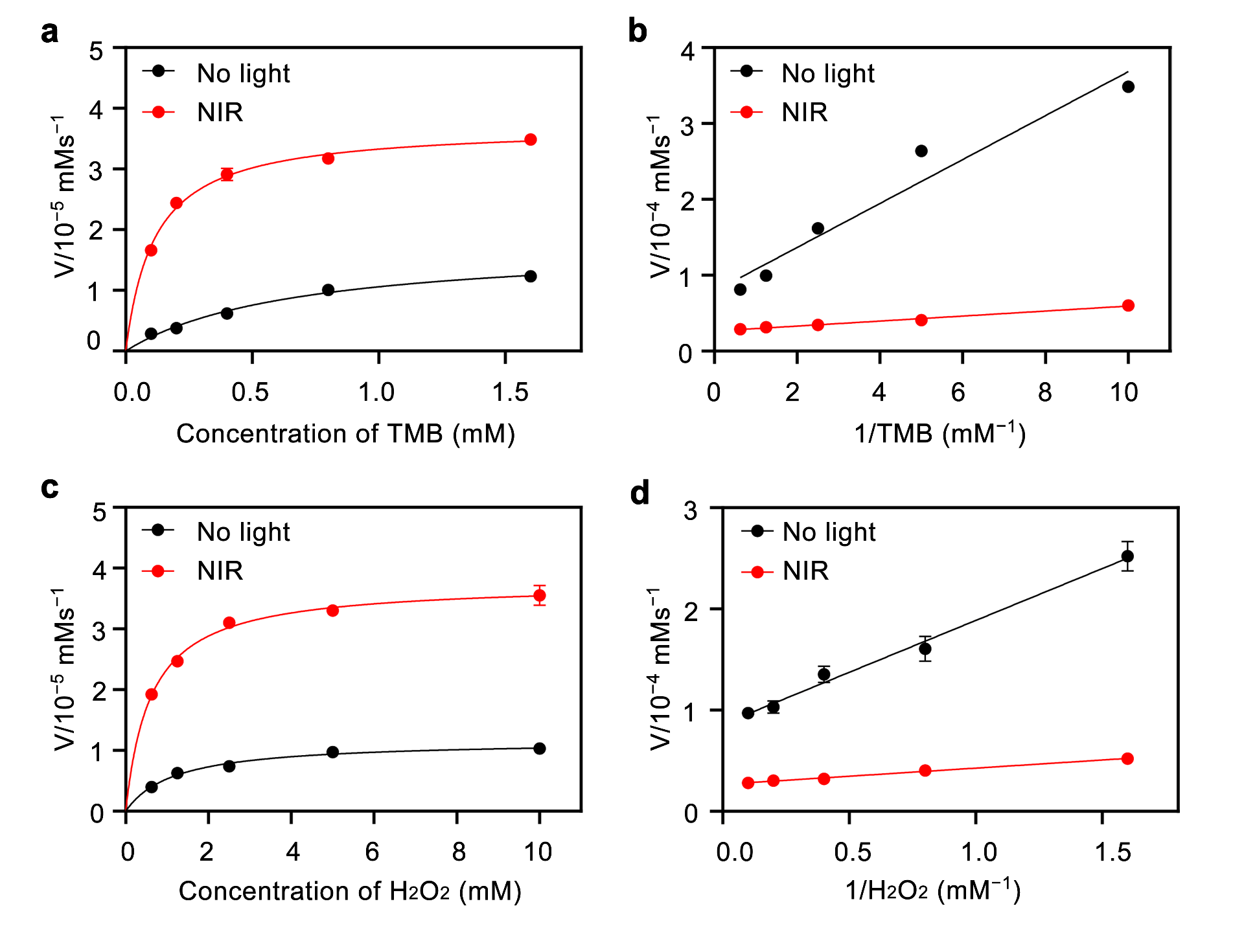


**Fig. S12** Steady-state kinetics analysis of ePt-Au NBPs with or without NIR-II irradiation (0.6 W cm^-2^). (a) Steady-state kinetics measurement of TMB. (b) Double-reciprocal plot of TMB. (c) Steady-state kinetics measurement of H_2_O_2_. (d) Double-reciprocal plot of H_2_O_2_.


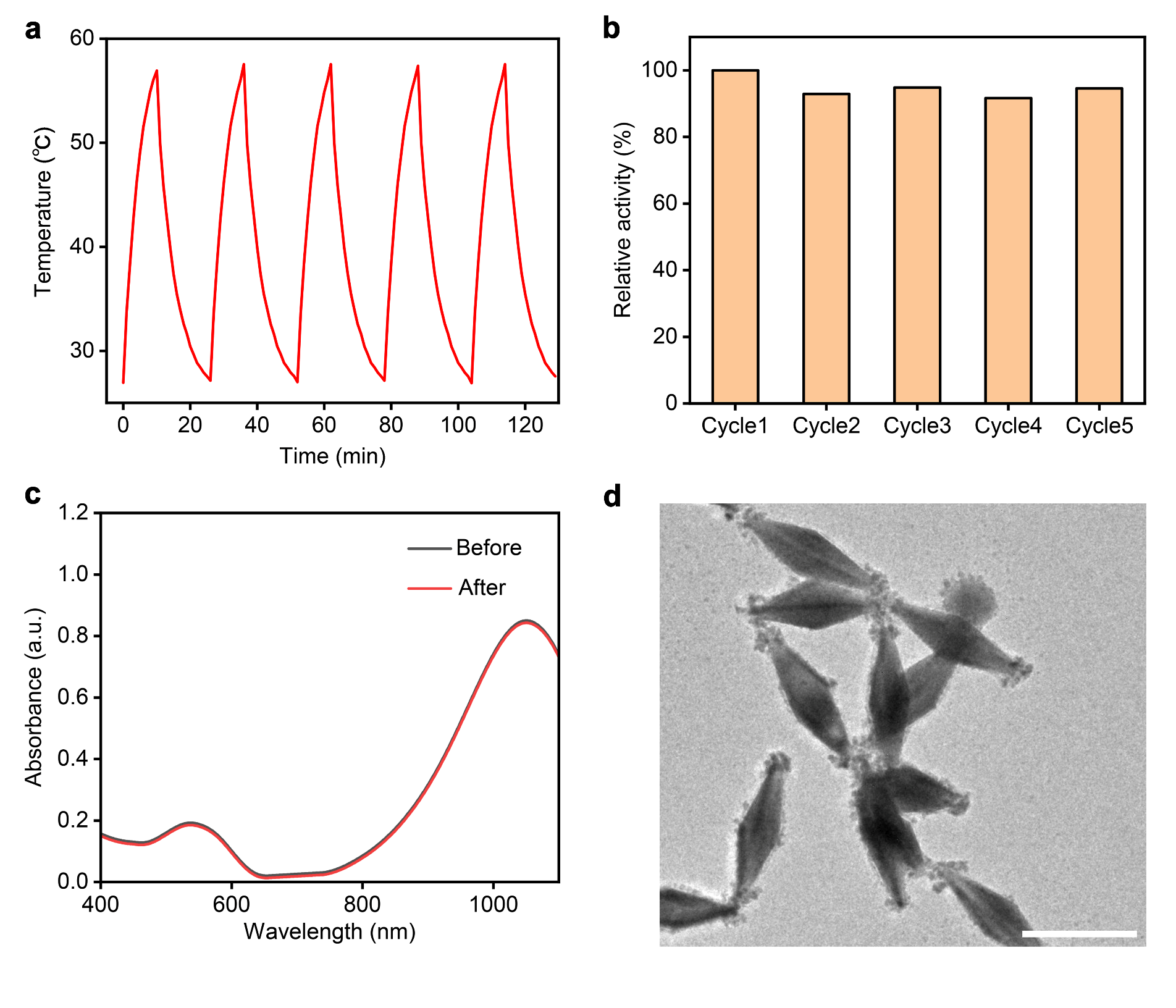


**Fig. S13** Evaluation of the stability of ePt-Au NBPs after NIR-II laser irradiation (0.6 W cm^-2^). (a) Photothermal stability. (b) Photocatalytic stability. (c) UV-Vis-NIR absorption spectra of ePt-Au NBPs before and after NIR-II laser irradiation. (d) TEM image of ePt-Au NBPs after NIR-II laser irradiation (scale bar: 100 nm).


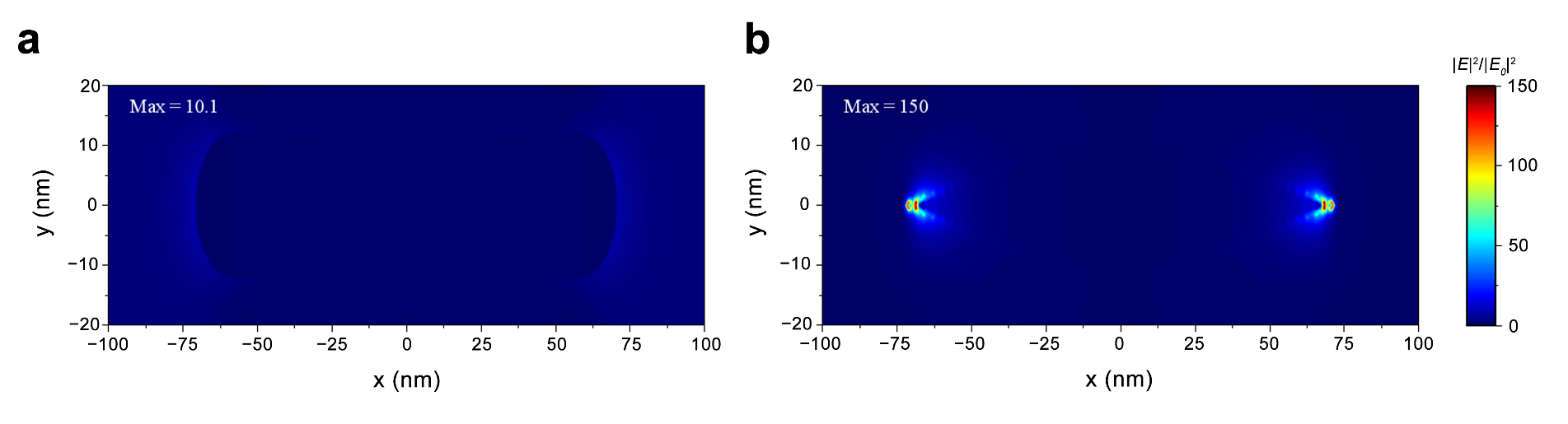


**Fig. S14** EM field distribution of Au NRs (a) and Au NBPs (b) simulated by FDTD.


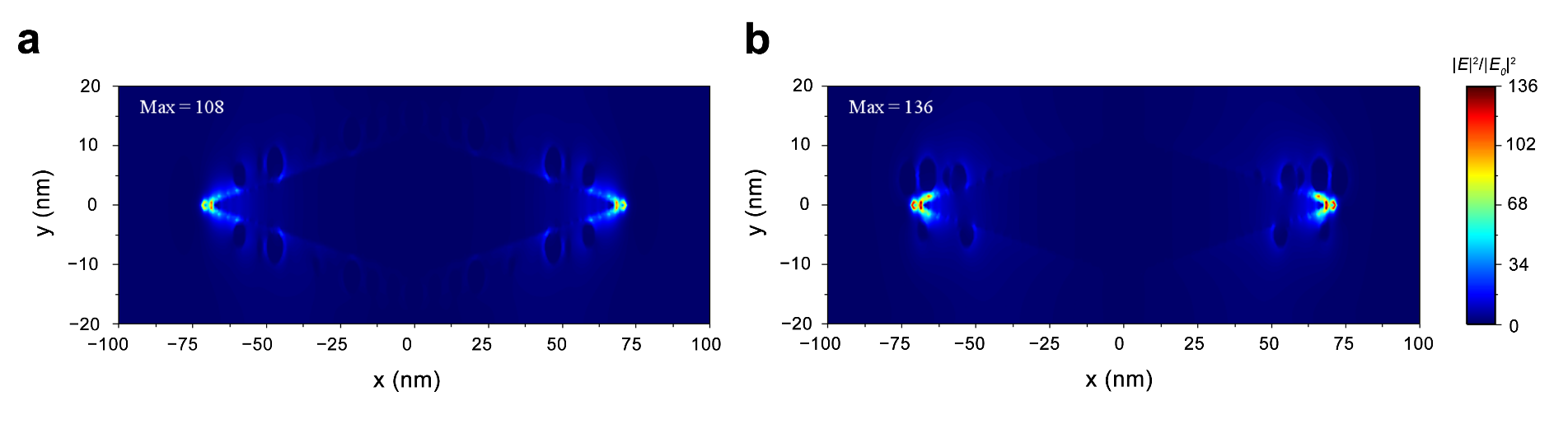


**Fig. S15** EM field distribution of aPt-Au NBPs (a) and ePt-Au NBPs (b) simulated by FDTD.


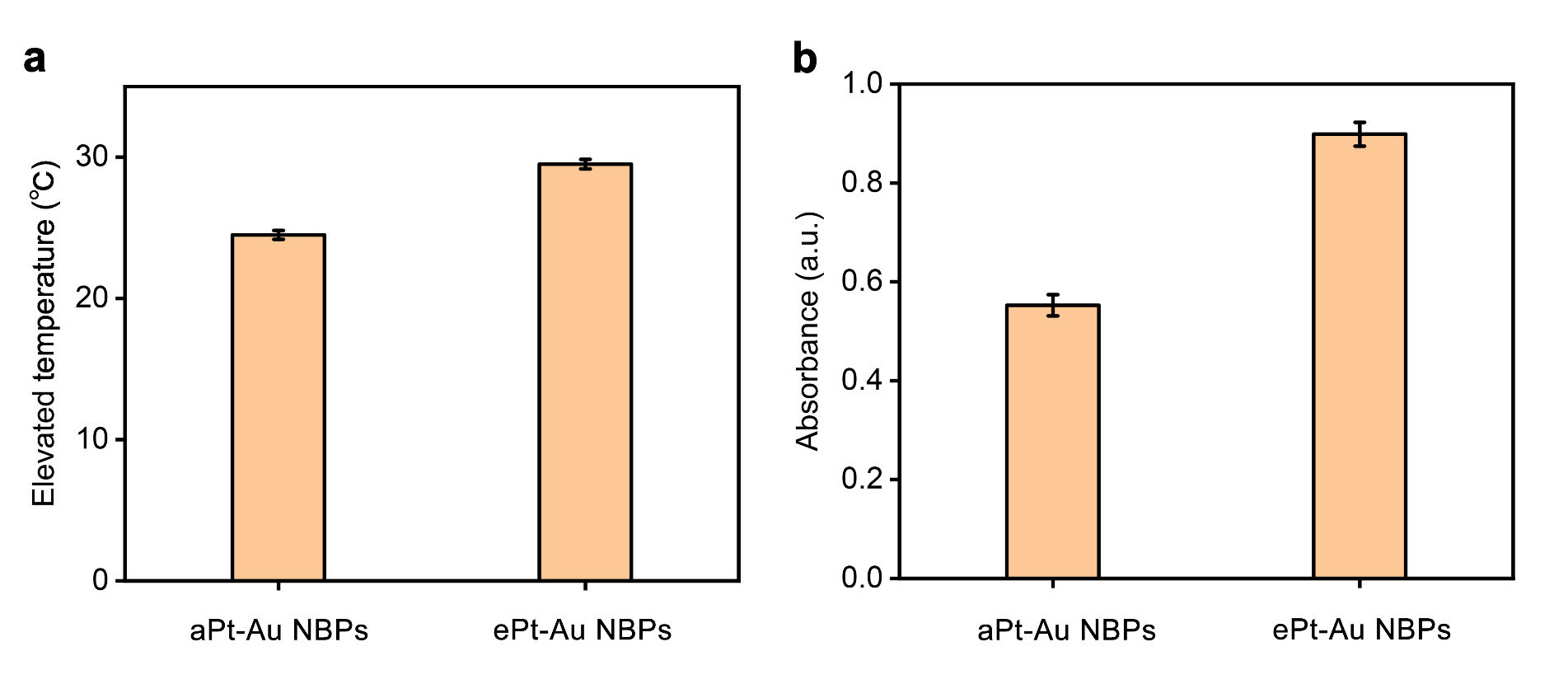


**Fig. S16** Photothermal (a) and photocatalytic activity (b) of aPt-Au NBPs and ePt-Au NBPs solution under NIR-II laser irradiation (0.6 W cm^-2^).


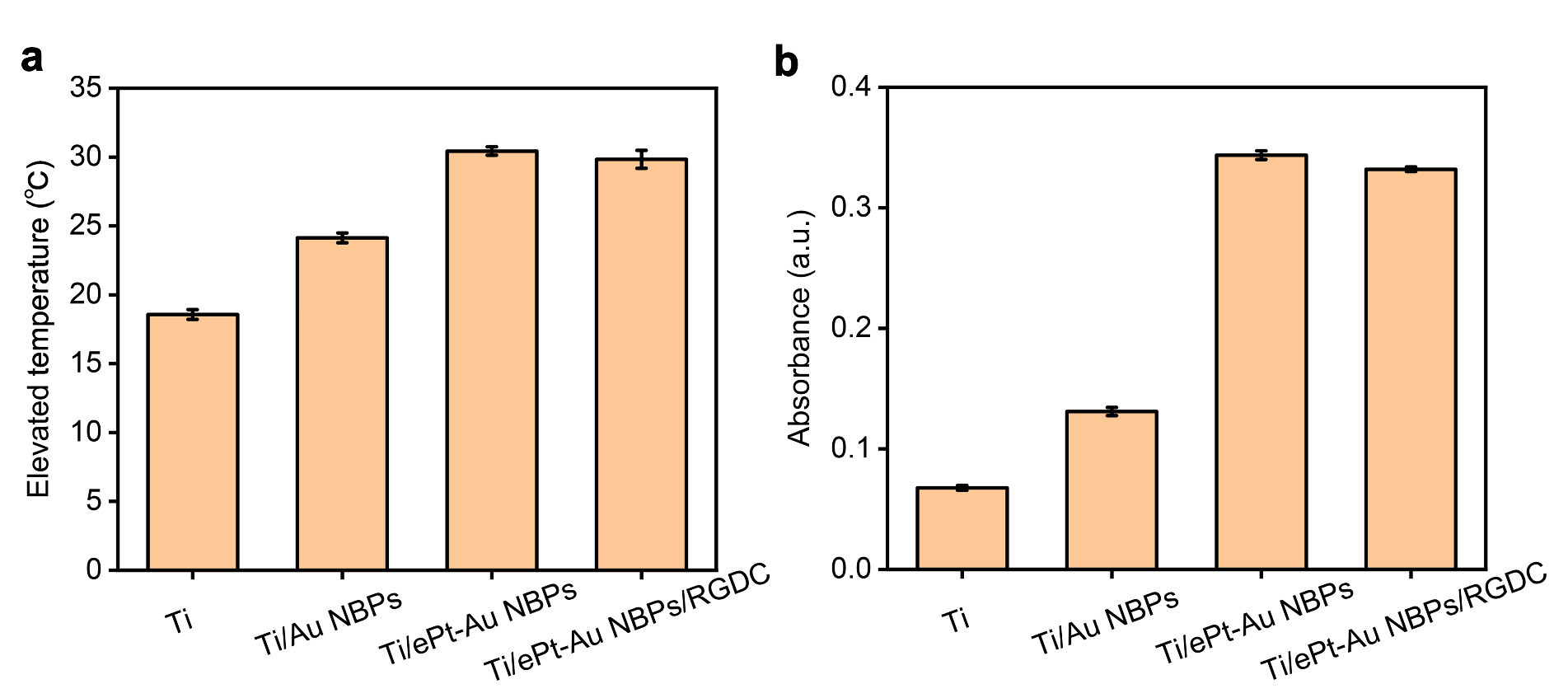


**Fig. S17** Temperature elevation statistics (a) and peroxidase-like activity statistics (b) of different implants (0.6 W cm^-2^).


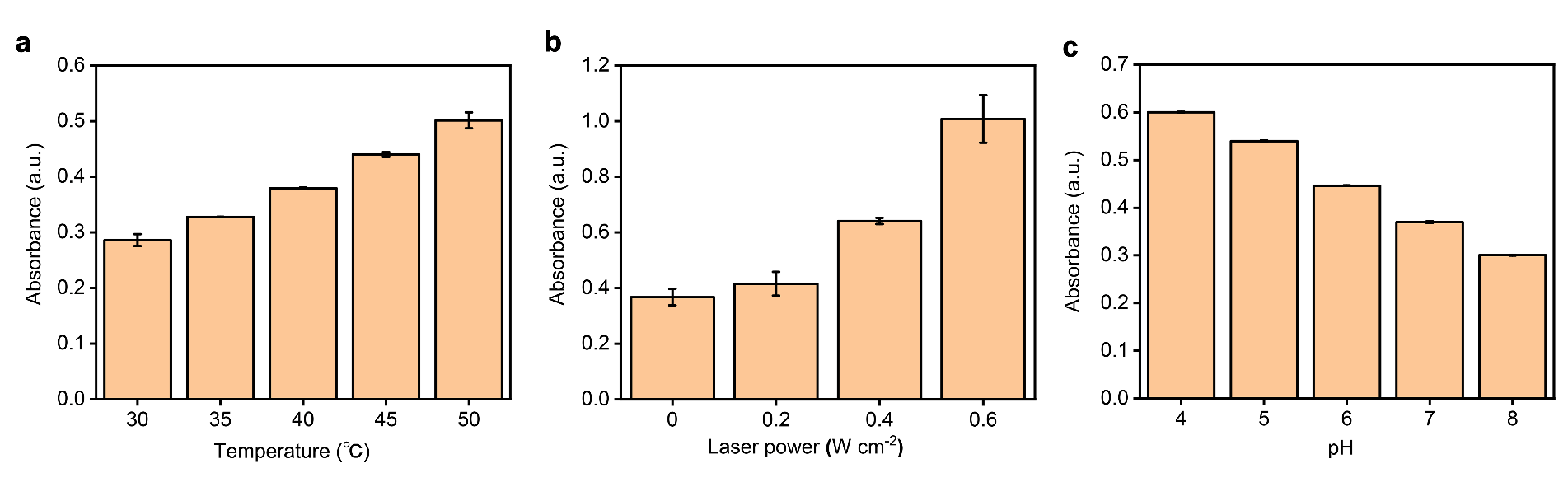


**Fig. S18** Factors influencing the peroxidase-like activity of Ti/ePt-Au NBPs/RGDC. (a) Temperature. (b) Laser intensity. (c) pH.


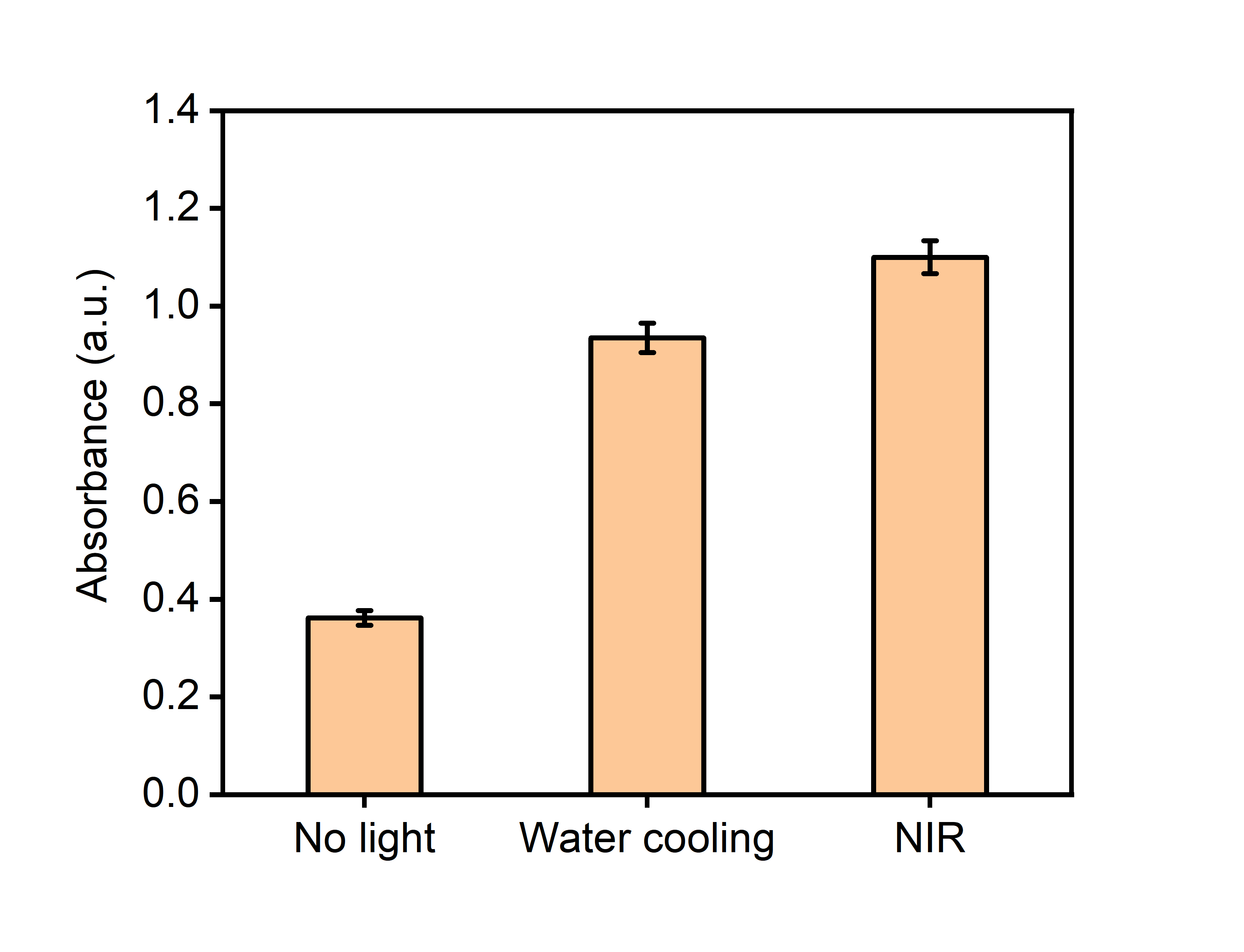


**Fig. S19** Comparison of the effects of photocatalysis and photothermal effects on enzymatic activity (0.6 W cm^-2^).


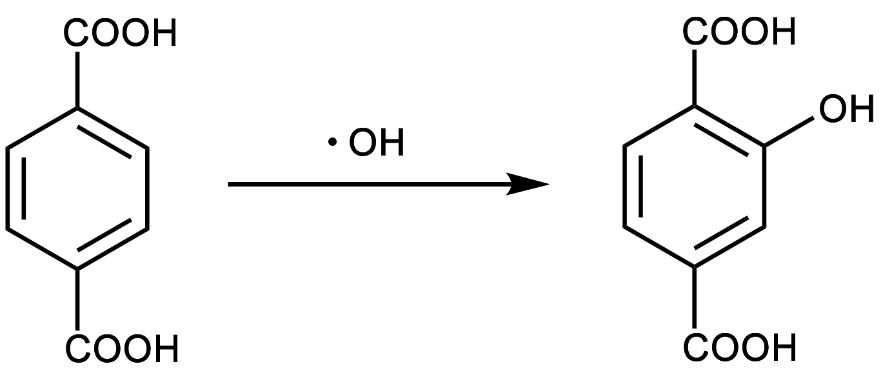


**Fig. S20** Reaction of TA with •OH.


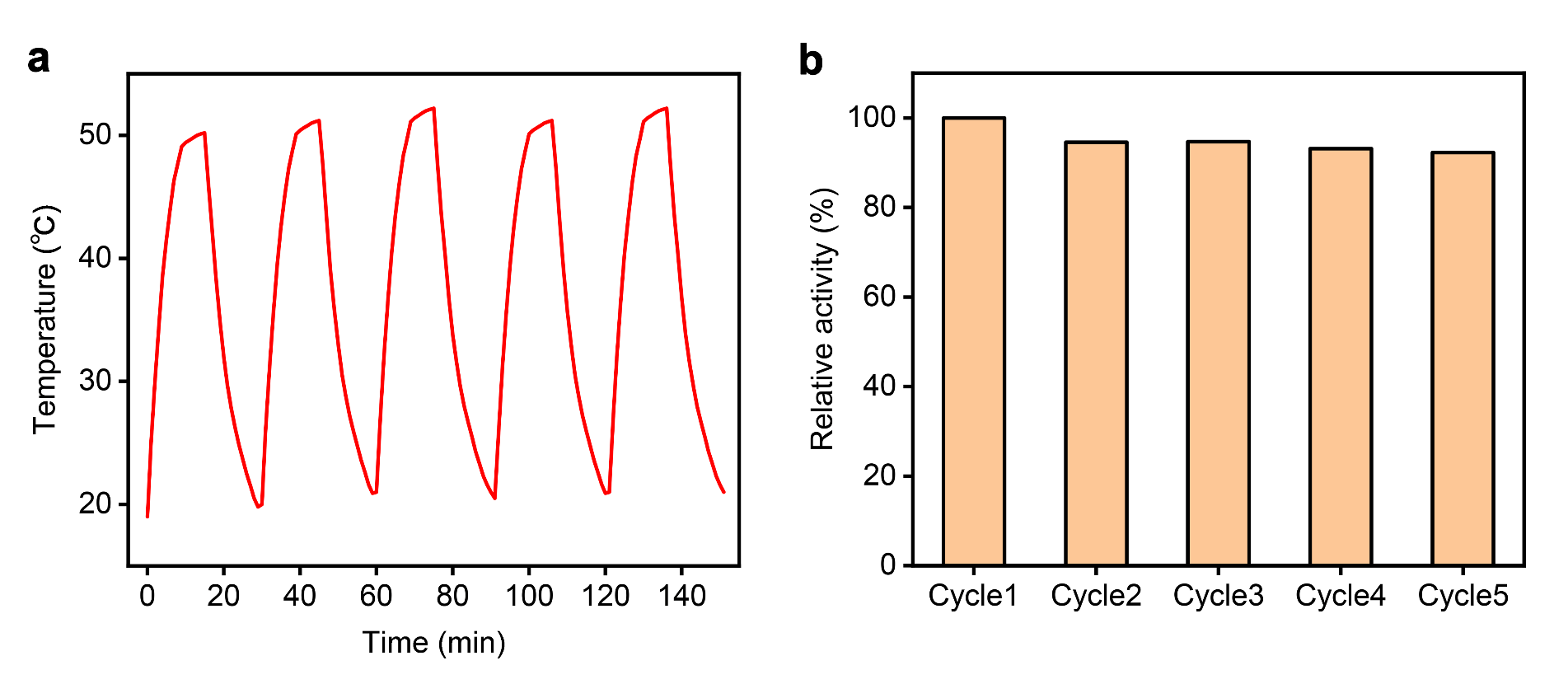


**Fig. S21** Evaluation of the stability of Ti/ePt-Au NBPs/RGDC after NIR-II laser irradiation (0.6 W cm^-2^). (a) Photothermal stability. (b) Photocatalytic stability.


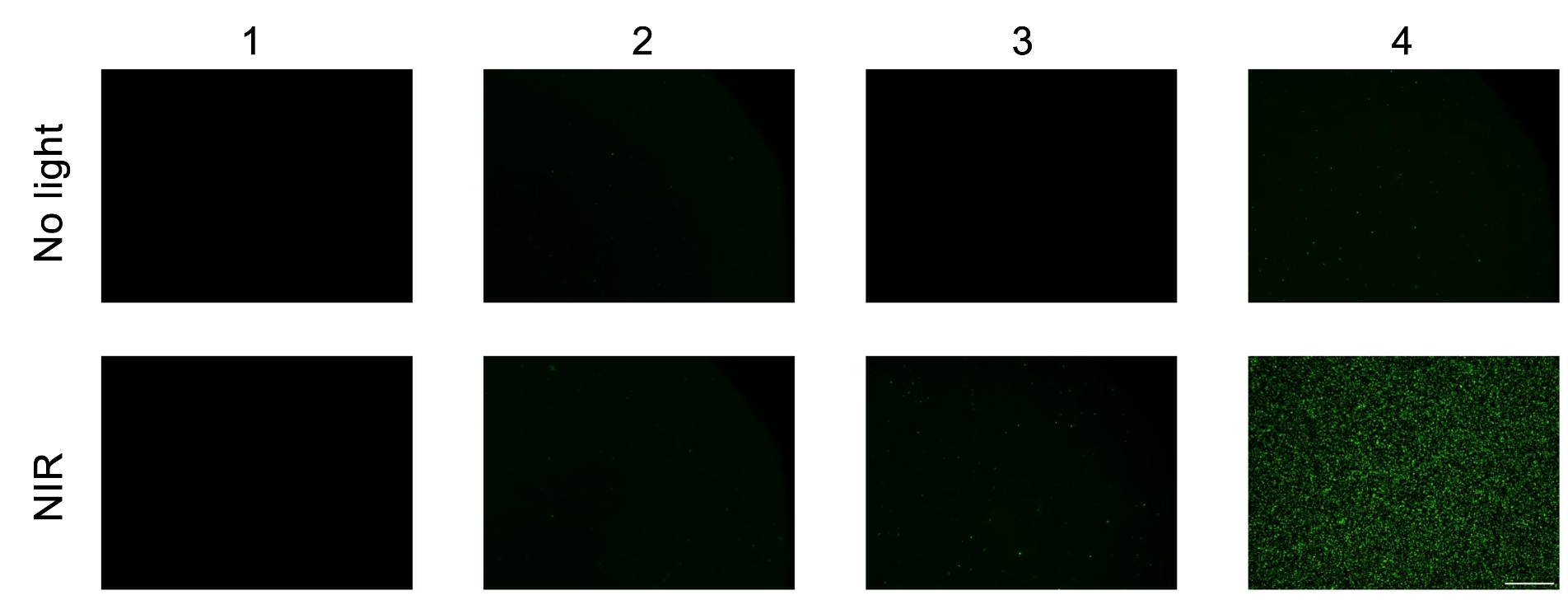


**Fig. S22** Fluorescence images of bacteria treated with different conditions showing the production of ·OH (0.6 W cm^-2^) (scale bar: 100 μm). (1) Ti, (2) Ti + H_2_O_2_, (3) Ti/ePt-Au NBPs/RGDC, (4) Ti/ePt-Au NBPs/RGDC + H_2_O_2_.


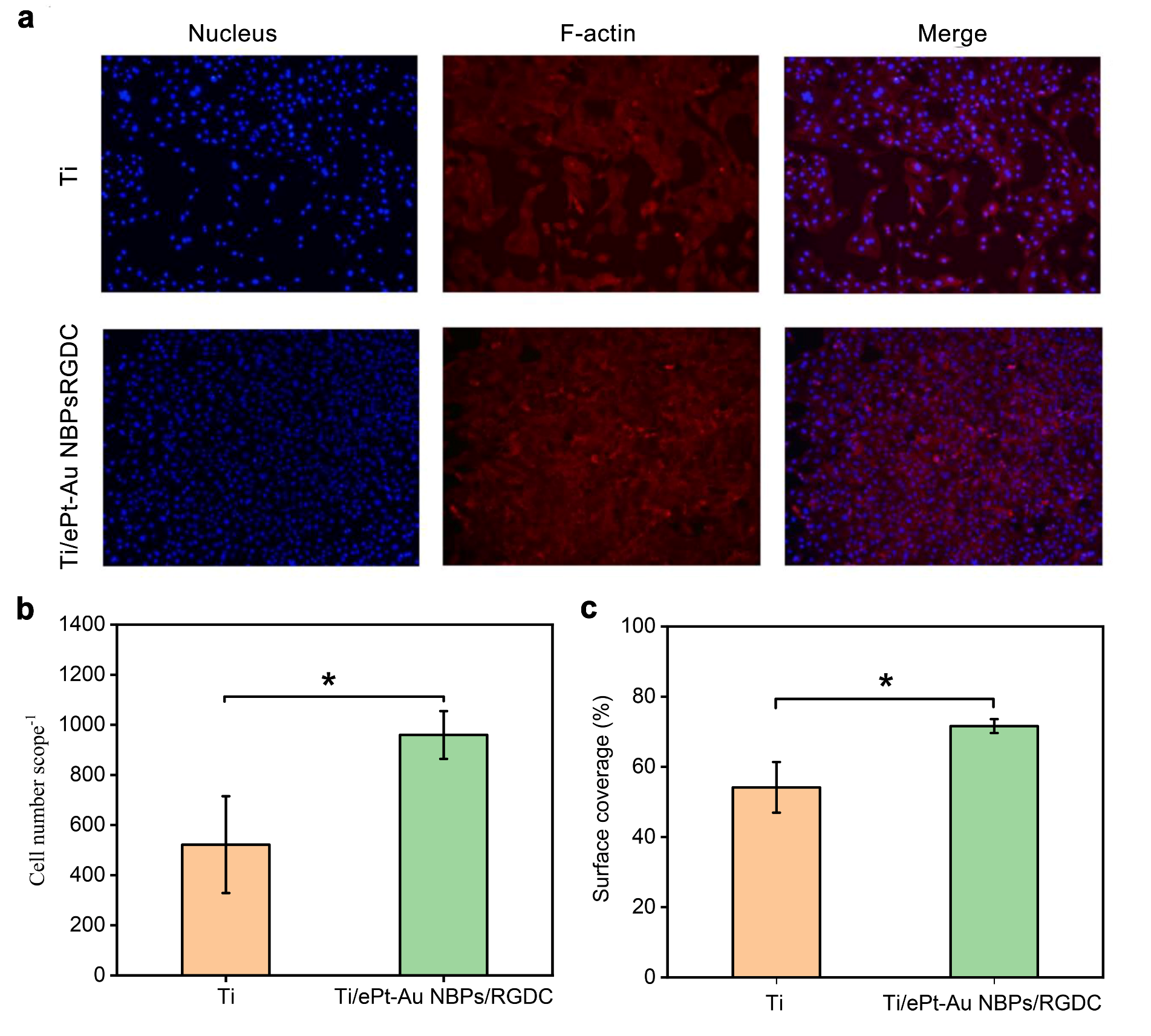


**Fig. S23** Assessment of adhesion capacity of implants. (a) Morphology of MC3T3-E1 cells after co-culture with implants for 24 h. (b) Quantification of cell adhesion count. (c) Quantification of cell adhesion area. (*p < 0.05, **p < 0.01 and ***p < 0.001).


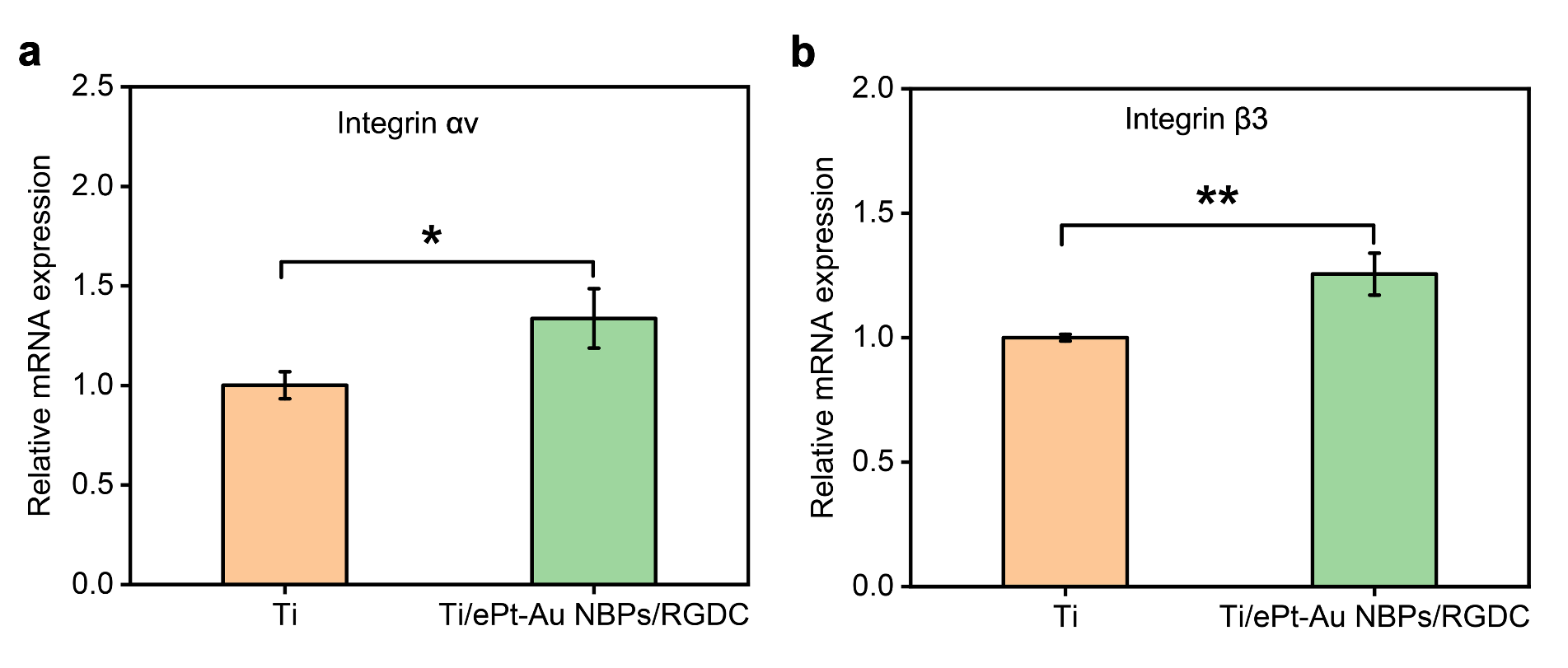


**Fig. S24** Relative gene expression levels of adhesion genes. (a) Integrin αv. (b) Integrin β3. (*p < 0.05, **p < 0.01 and ***p < 0.001).


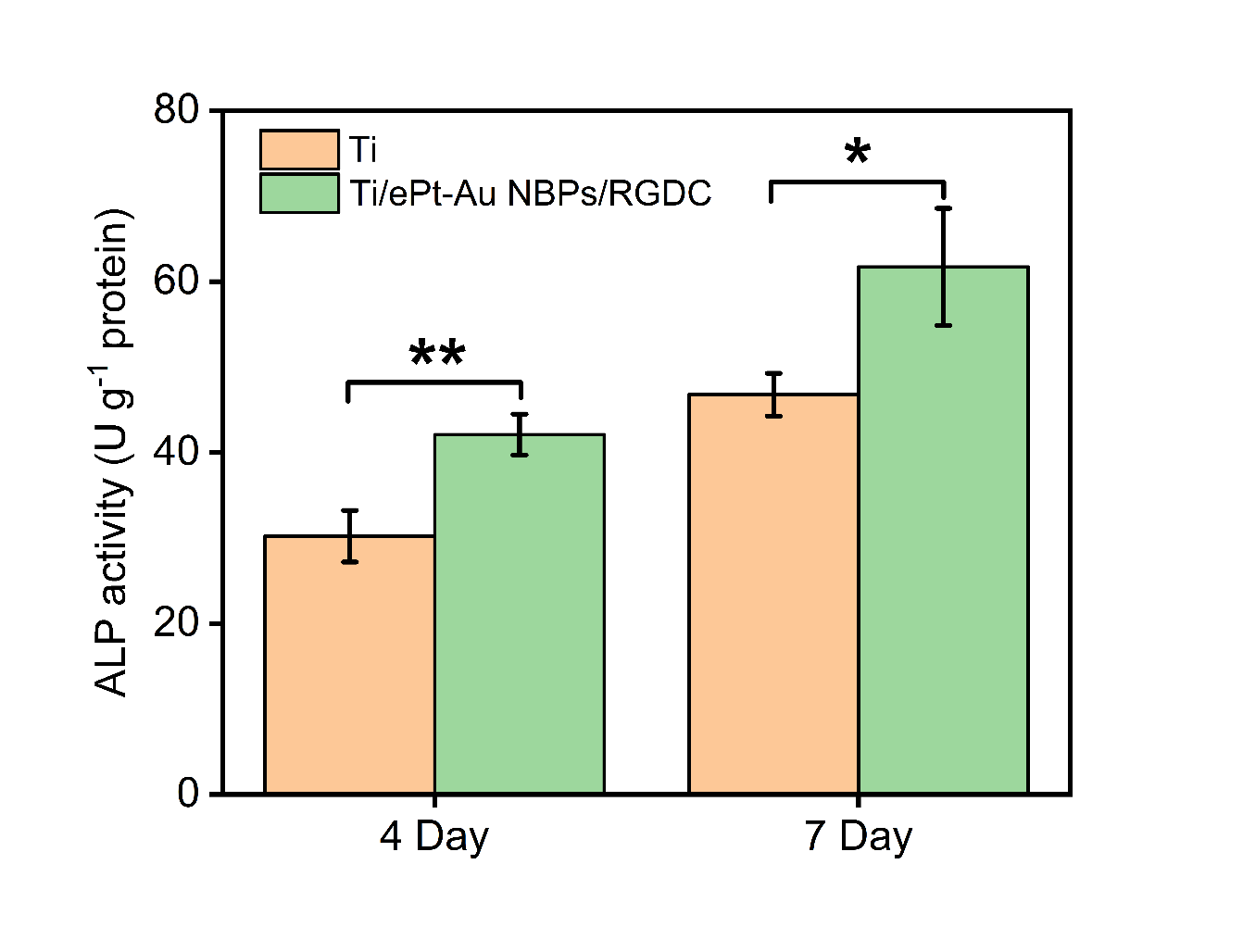


**Fig. S25** ALP activity of MC3T3-E1 cells on different implants. (*p < 0.05, **p < 0.01 and ***p < 0.001).


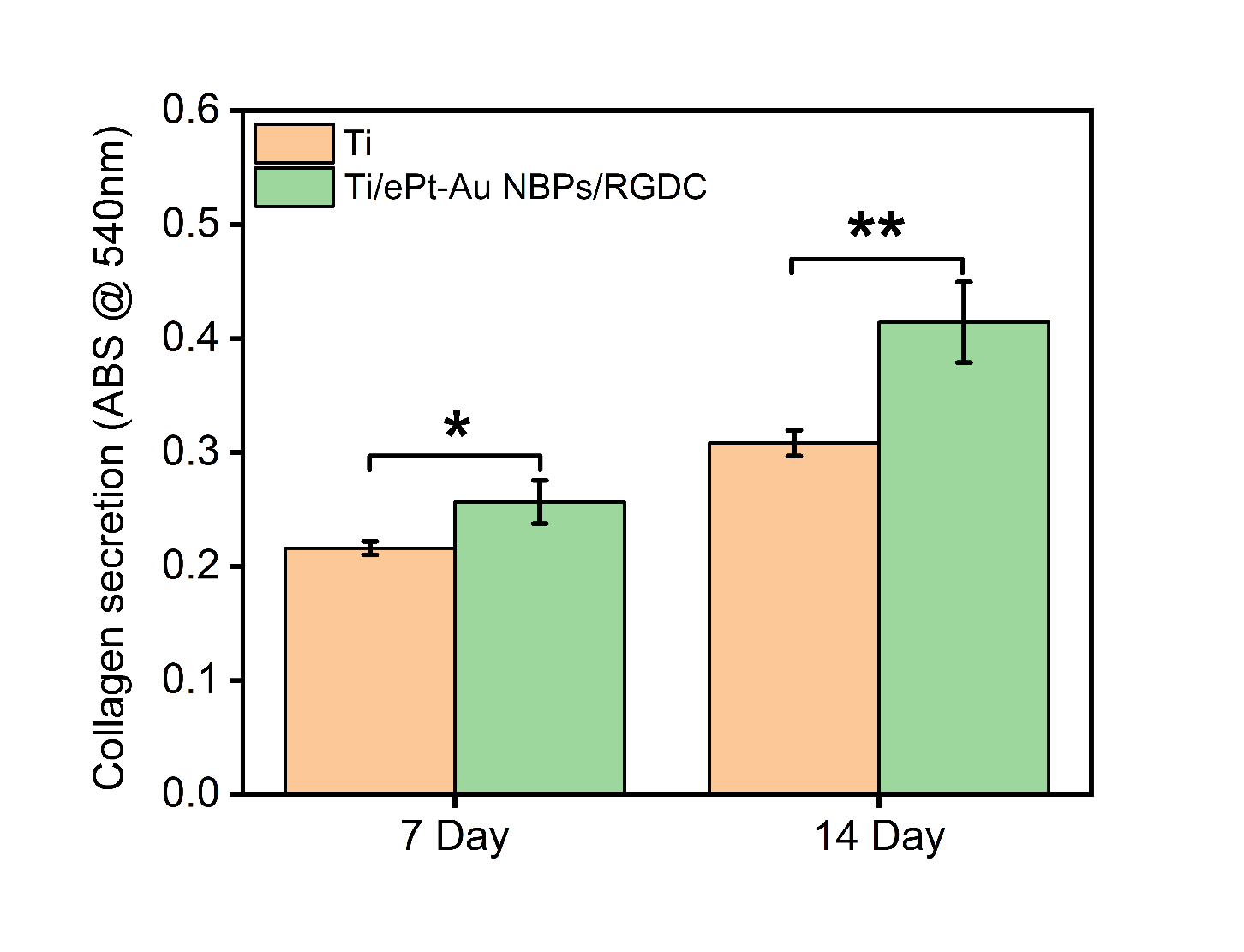


**Fig. S26** Collagen secretion level of MC3T3-E1 cells on different implants. (*p < 0.05, **p < 0.01 and ***p < 0.001).


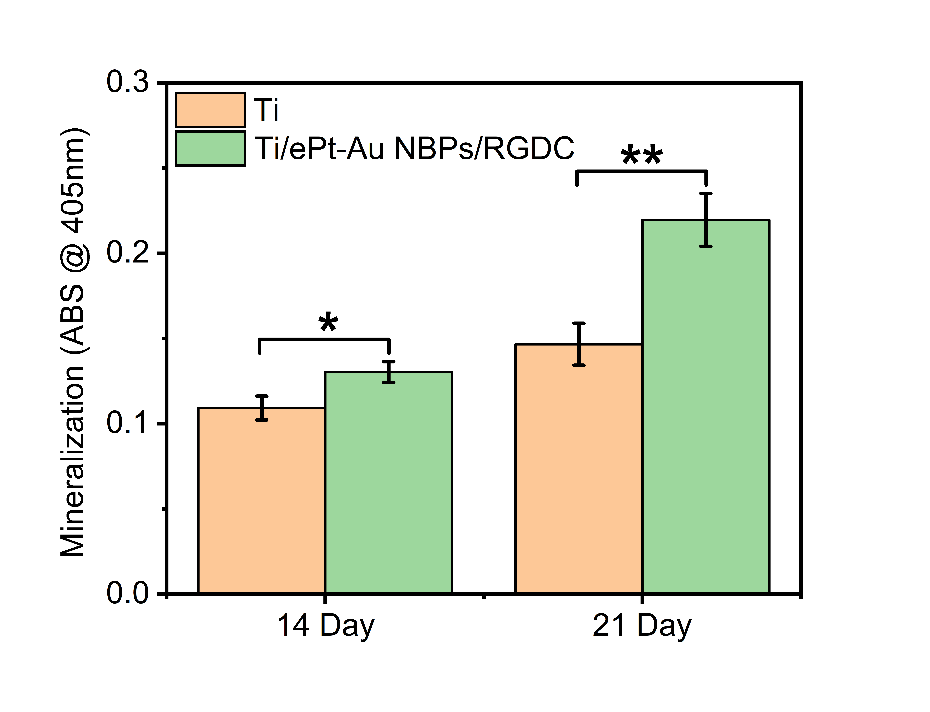


**Fig. S27** ECM mineralization level of MC3T3-E1 cells on different implants. (*p < 0.05, **p < 0.01 and ***p < 0.001).


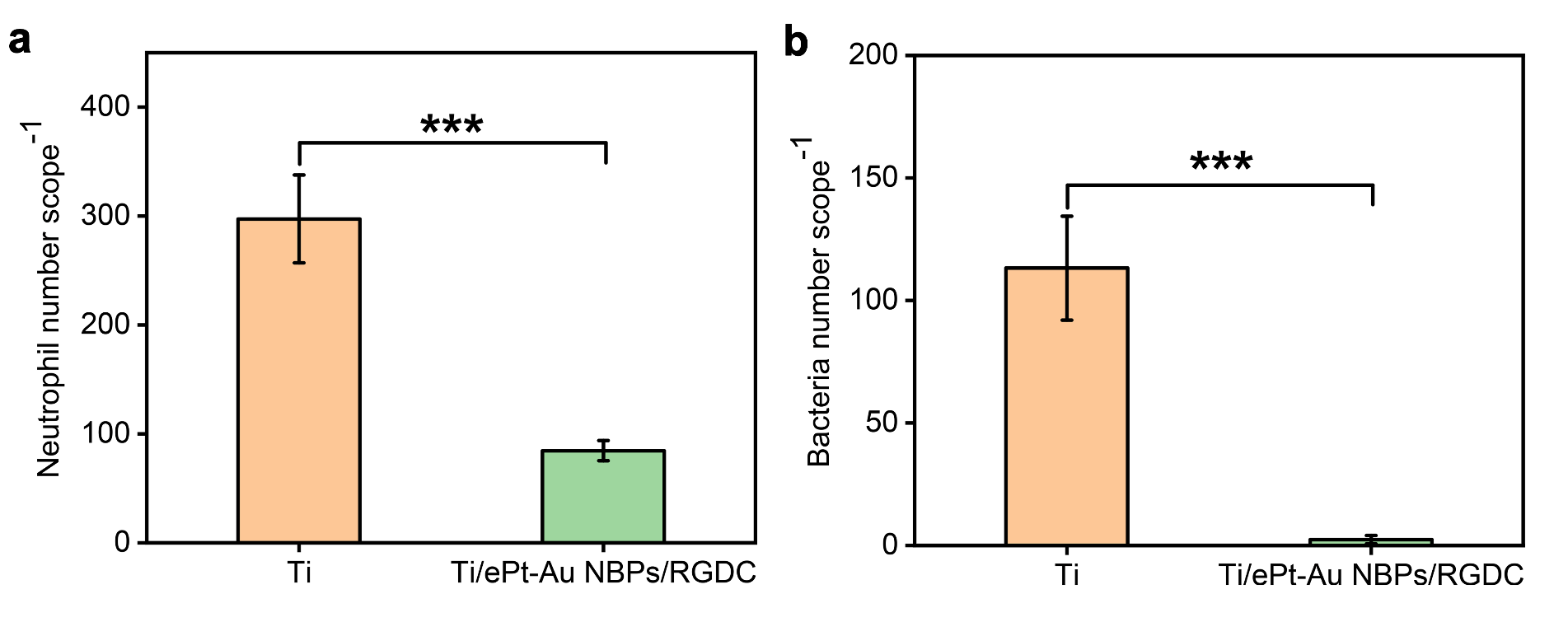


**Fig. S28** Statistics of inflammatory infiltration and bacterial infection in the soft tissue surrounding the implants. (a) Number of neutrophils in the soft tissue surrounding the implants. (b) Number of bacteria in the soft tissue surrounding the implants. (*p < 0.05, **p < 0.01 and ***p < 0.001).


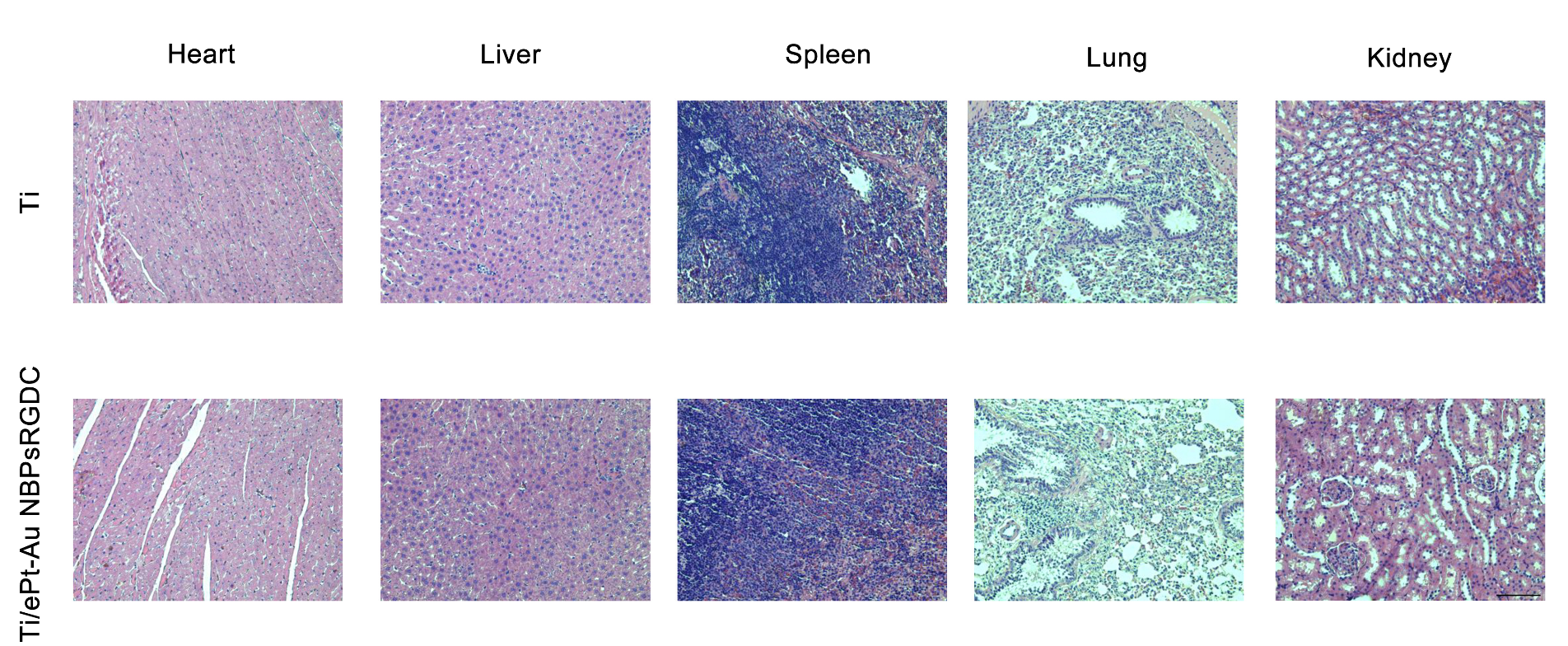


**Fig. S29** H&E staining of major organs (heart, liver, spleen, lung, and kidney) (scale bar: 100 μm).


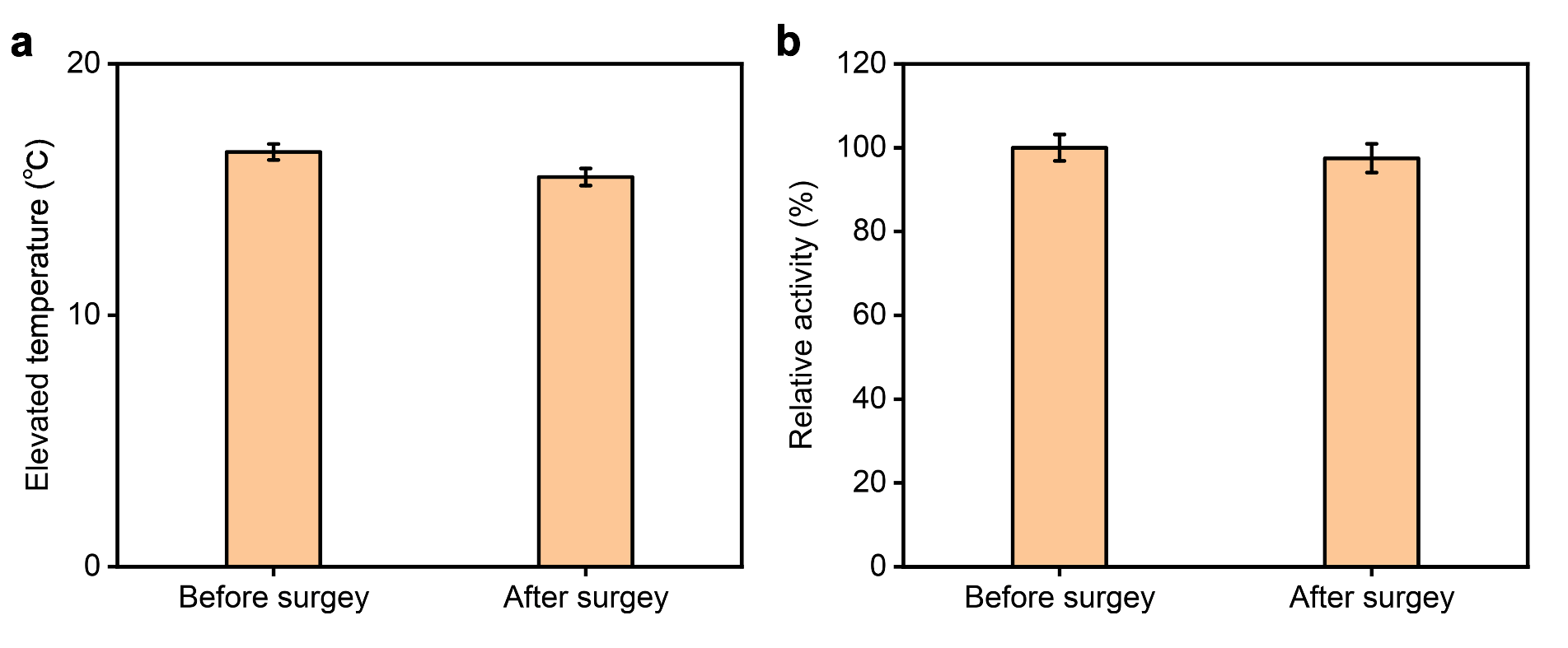


**Fig. S30** Detection of photothermal (a) and photocatalytic (b) stability before and after implantation surgery (0.6 W cm^-2^).

**Table S1.** The primer sequences for RT-qPCR analysis.

| Primers | Sequence (5′-3′) |
| --- | --- |
| GAPDH-F | AAATGGTGAAGGTCGGTGTGAAC |
| GAPDH-R | CAACAATCTCCACTTTGCCACTG |
| Integrin αv-F | CGAAGCCTTAGCAAGACTGTCCTG |
| Integrin αv-R | CGAAGACCAGCGAGCAGTTGAG |
| Integrin β3-F | GGAAGGCTGGCAGGCATTGTC |
| Integrin β3-R | ATGGTAGTGGAGGCAGAGTAGTGG |
| ALP-F | GCCCTCCAGATCCTGACCAA |
| ALP-R | GCAGAGCCTGCTGGTCCTTA |
| Runx2-F | AGGAATGCGCCCTAAATCACT |
| Runx2-R | ACCCAGAAGGCACAGACAGAAG |
| COLI-F | GCTCCTCTTAGGGGCCACT |
| COLI-R | CCACGTCTCACCATTGGGG |
| OCN-F | GAACAGACAAGTCCCACACAGC |
| OCN-R | TCAGCAGAGTGAGCAGAAAGAT |
